# Nanogels as Novel Nanocarrier Systems for Efficient Delivery of CNS Therapeutics

**DOI:** 10.3389/fbioe.2022.954470

**Published:** 2022-07-19

**Authors:** Yunhan Zhang, Zhulin Zou, Shuang Liu, Shengjie Miao, Haiyan Liu

**Affiliations:** Department of Anatomy, College of Basic Medicine Sciences, Jilin University, Changchun, China

**Keywords:** nanotechnology, nanogel, CNS diseases, blood-brain barrier, smart drug release

## Abstract

Nanogels have come out as a great potential drug delivery platform due to its prominently high colloidal stability, high drug loading, core-shell structure, good permeation property and can be responsive to environmental stimuli. Such nanoscopic drug carriers have more excellent abilities over conventional nanomaterials for permeating to brain parenchyma *in vitro* and *in vivo*. Nanogel-based system can be nanoengineered to bypass physiological barriers via non-invasive treatment, rendering it a most suitable platform for the management of neurological conditions such as neurodegenerative disorders, brain tumors, epilepsy and ischemic stroke, etc. Therapeutics of central nervous system (CNS) diseases have shown marked limited site-specific delivery of CNS by the poor access of various drugs into the brain, due to the presences of the blood-brain barrier (BBB) and blood-cerebrospinal fluid barrier (BCSFB). Hence, the availability of therapeutics delivery strategies is considered as one of the most major challenges facing the treatment of CNS diseases. The primary objective of this review is to elaborate the newer advances of nanogel for CNS drugs delivery, discuss the early preclinical success in the field of nanogel technology and highlight different insights on its potential neurotoxicity.

## Introduction

Neurological diseases and disorders are considered significant challenges to the human health. According to global statistics, more than 1.5 billion people, arguably a quarter of the world’s population suffer from CNS diseases (Palmer, 2010; Srikanth and Kessler, 2012; Soni et al., 2016a). A wide spectrum of therapeutic agents, e.g., nucleic acids, polypeptides, proteins and antisense drugs, have been promoted to alleviate CNS diseases, but the majority of these agents are unable to enter the brain parenchyma noninvasively due to a series of challenges. Site-specific delivery of the drugs is one of the most significant challenges due to restrictions imposed by two biochemical barriers, primarily by BBB. To treat CNS diseases, the most lethal and disabling diseases in the world, researchers have attempted to explore the novel treatment strategies using multiple perspectives ranging from more established efforts, such as the disruption of the BBB, the alteration of BBB permeability, and lipidization of water-soluble drugs, to the newer methods such as the development of nanoenabled drug delivery systems.

Nanotechnology is one of the strategies that provide a greater possibility to meet the requirements associated with high doses of the CNS drugs. Nanocarriers for the management of CNS diseases mainly include polymeric nanoparticles, solid lipid nanoparticles (SLNs), lipid nanocapsules, albumin nanoparticles, liposomes, dendrimers, nanoemulsions, hydrogels and nanogels, etc. (Date et al., 2007; Sun et al., 2012; Cojocaru et al., 2020; Bhia et al., 2021) However, these agents have the following inherent limitations in preclinical applications: 1) liposomes have poor stability and dispersibility; 2) polylactic acid-glycolic acid copolymer NPs but suffer from burst release problems; 3) chitosan nanoparticles have poor dispersibility when used in humans and potential biotoxicity; 5) inorganic-based carriers still cannot achieve biocompatibility; and 6) superparamagnetic iron oxide that contain choline has been mainly been fabricated for *in vivo* imaging studies. These limitations may render drugs unable to treat CNS diseases with ideal effects. Table 1 summarizes several classes of the drug nanocarriers and their limitations for CNS therapy.

**TABLE 1:** A brief list of properties for nanocarriers used in the field of CNS therapeutics.

Nanocarriers	Type of materials	Advantages/limitations	Drug/agent delivered	References
Polymer NPs	PACA	BBB penetration increased, less immunogenic reaction/High potential toxicity risk	Methotrexate	Tian et al. (2011)
	PLA		Temozolomide	Javed et al. (2021)
	PLGA		Piperine	Guo et al. (2017)
Dendrimers	PAMAM	Easy functionalization/Ligand-targeted conjugation, suitable for drug entrapment/ limited synthetic route, hematological toxicity	*o*-phenylene	(Medina and El-Sayed, 2009; Zain-ul-Abdin et al., 2017)
	PPI		Diamine	
	poly(lysine)		Lamivudine	(Dutta and Jain, 2007; Pargoo et al., 2021)
			Doxorubicin	Zhang et al. (2014)
Solid lipid NPs (SLNs)	Sphingomyelin	High BBB permeability, increased drug release, drug levels rise in the brain/Rapid clearance, neurotoxicity from surfactant	siRNA	(Jin et al., 2011; Bae et al., 2013; Buyukkoroglu et al., 2016)
	Stearic acid		Doxorubicin	(Tangpong et al., 2007; Battaglia et al., 2014)
	Cholesterol		Insulin	Kuo and Shih-Huang, (2013)
	PC		Azidothymidine	Pottoo et al. (2020)
Micelles	Block copolymeric	Drug permeability increased, enhanced oral bioavailability/Low encapsulation efficiency of drugs	Pilocarpine	Meng et al. (2020)
	Micelles		Paclitaxel	Zhang et al. (2012)
	Core-shell micelles		Vinblastine	Wong et al. (2012)
Emulsion	Edible oil	High biocompatibility, drugs uptake increased/Heat decomposition, uncontrollable release, storage instability	Saquinavir	Mahajan et al. (2014)
	PUFA		Coenzyme Q10	Pastor-Maldonado et al. (2020)
	Flaxseed Oil		Cyclosporin A	Francischi et al. (1997)
Liposome	PC/cholesterol PC/PG Phospholipid/ cholesterol	Large drug loading capacity, extended half-life of the drug/Low distribution in tumor tissue, poor stability	Daunorubicin Cisplatin Amikacin	Fassas and Anagnostopoulos, (2005) Huo et al. (2012) Khan and Chaudary, (2020)
Nanogel	PEG-PLA	Facile synthesis, extended systemic exposure, enhanced bioavailability, respond to external stimuli, high distributed in lesion tissue	Methotrexate	(Pourtalebi Jahromi et al., 2018; Pourtalebi Jahromi et al., 2019)
	CHP		Nano-NRTI	(Vinogradov et al., 2010; Gerson et al., 2014; Warren et al., 2015)
	PEG-PEI		5-fluorouracil Antisenseoligonucleotides	Zhou et al. (2013)
	PEG-PLA			(Vinogradov et al., 2004; Wang and Wu, 2017)

**FIGURE 1:**
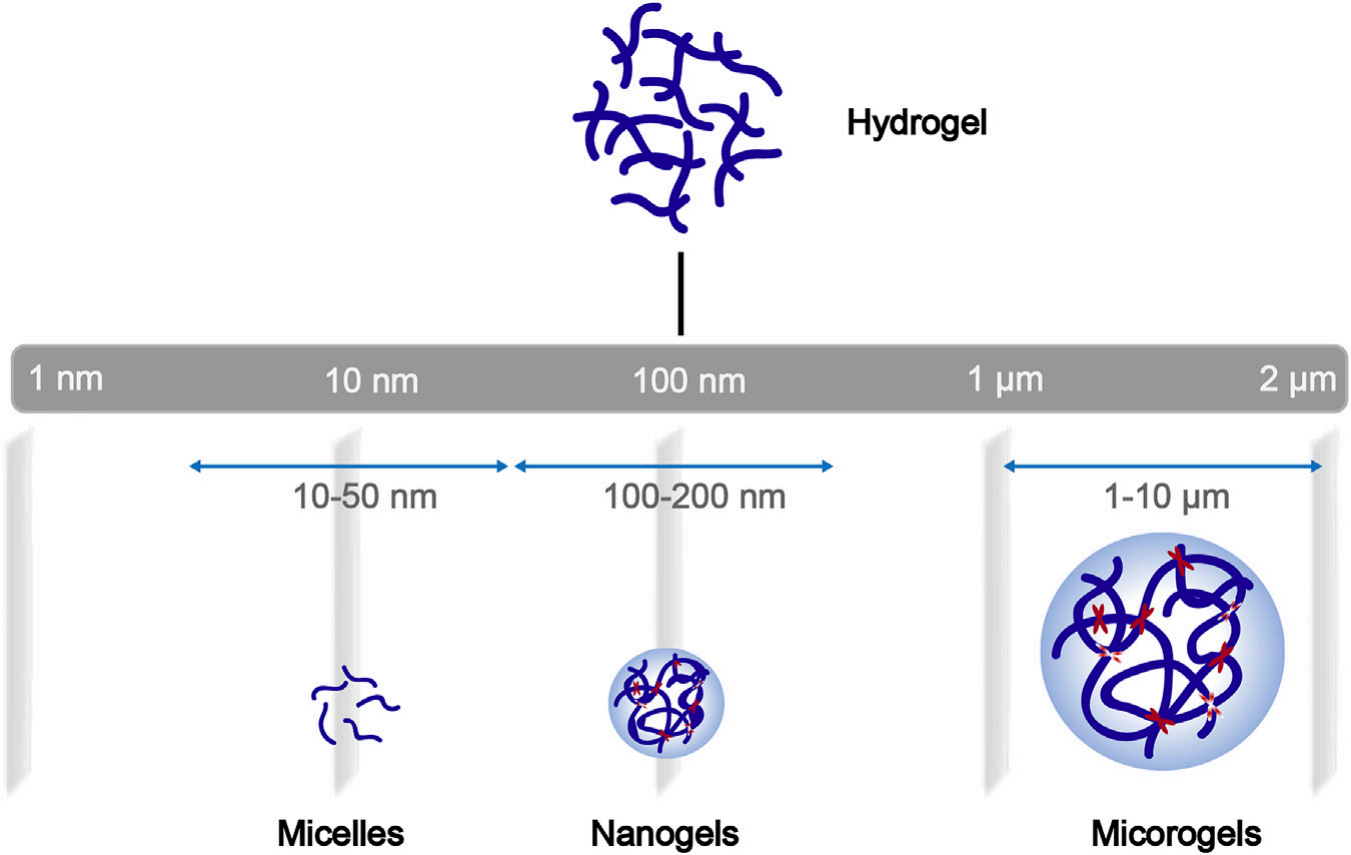
General schematization of the network construction of hydrogels and derivatives of different sizes.

**FIGURE 2:**
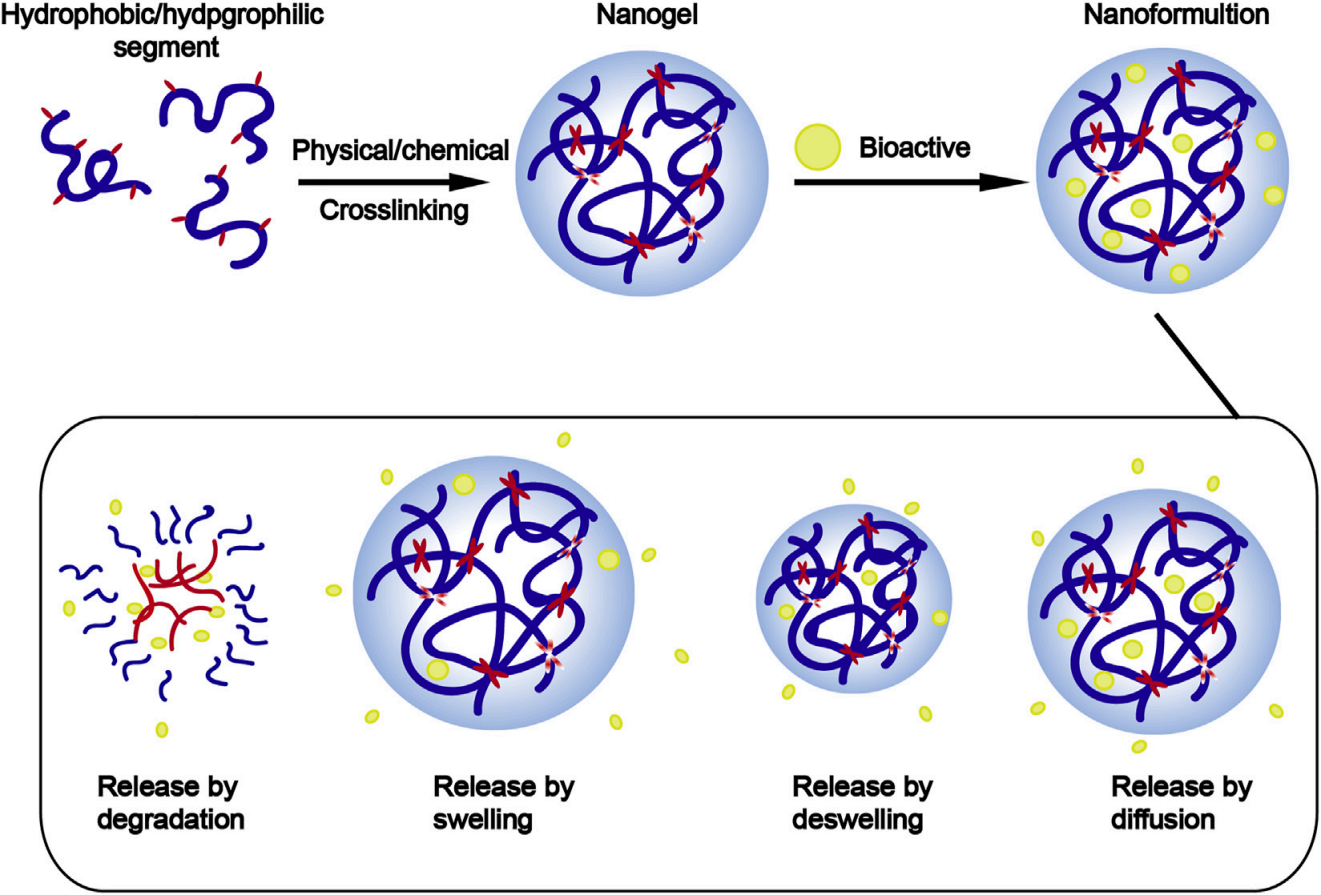
Schematic representation of the synthesis and the release behavior of nanogels.

Among these nanotechnologies, the transition from hydrogels to nanogels offers new opportunities to achieve a systemic controlled-release drug delivery platform at the cellular level. Traditional hydrogels exhibit limited BBB penetration ability due to their microstructure, but this obstacle can be overcome by designing nanogels as nanosized colloidal particles (Figure 1). (Chen et al., 2021) Nanogels are the nanoscale hydrogel materials consisting of crosslinked polymer networks, which can be easily synthesized by chemical or physical routes (Figure 2). (Yin et al., 2008; Merino et al., 2015; Neamtu et al., 2017) Such nanocarrier systems are characterized by excellent ability to cross the BBB and accomplish site-specific delivery of drugs due to high water retention capacity of the hydrogels, their tunable shape, amphiphilic behavior, surface modifiability, and especially to their biodegradability and safety. Currently, nanogel technologies are mainly manifested in the fields of disease diagnosis (medical imaging) and drug delivery. The latter application faces more challenges, especially for therapeutic drug delivery for brain diseases, due to complex physiological responses *in vivo* (Debele et al., 2016; Ma et al., 2017; Neamtu et al., 2017; Ali et al., 2021).

The present review provides insights into nanogels as an effective carrier system for CNS delivery in preclinical applications, the strategies to improve the BBB penetration, and the approaches that are close to clinical applications. The present article considers the major challenges for the nanogels, which remain despite certain advances in the design of CNS nanocarrier, to provide ideal plans for the clinical therapies of CNS diseases.

## Biological Barriers for CNS Drug Delivery

### The Blood-Brain Barrier

CNS homeostasis is strictly protected by two peripheral barriers, termed blood-brain barrier (BBB) and blood-cerebrospinal fluid barrier (BCSFB), which strictly regulate a series of the transport and metabolic processes to protect the brain from the periphery (Erickson and Banks, 2018; Ghersi-Egea et al., 2018; Kadry et al., 2020). These biological barriers, particularly the BBB pose the largest obstacle to nanocarriers for the delivery of various drugs into the CNS. In the absence of effective drug carriers, most macromolecules, such as proteins, oligonucleotides, nucleoside analogs, macromolecular drugs and more than 90% of small-molecule drugs cannot enter the brain through the BBB, which is the key bottleneck in CNS disease treatment (Pardridge, 2005; Wohlfart et al., 2012). These issues are the key bottleneck in the treatment of CNS diseases. Additionally, rapid drug clearance and the failure to achieve a steady release of the drugs in the brain tissue remain among the challenges for the treatment of this group of diseases (Misra et al., 2003; Furtado et al., 2018; Jain, 2020).

The BBB was defined for the first time by Paul Ehrlich in 1885 and is composed of unique CNS microvasculature, including tight junctions (TJs) and adherent junctions, which strictly regulate the metabolism of immune surveillance cells and the entry process of xenobiotics/endogenous materials (Hawkins and Davis, 2005; Engelhardt and Sorokin, 2009; Tietz and Engelhardt, 2015). The functional unit of the BBB is not only constituted by brain capillary endothelial cells (ECs), but also has close interactions with pericytes, perivascular astrocytes and nerve cells. In particular, the tight junction proteins claudins and occludin, which are expressed in brain microvascular cells, account for extremely high transendothelial electrical resistance (TEER, approximately 1,500–2,000 Ω cm^2^) of the BBB thus limiting the entry of the neurotherapeutic agents (Baeten and Akassoglou, 2011; Sá-Pereira et al., 2012; Molino et al., 2014; Girolamo et al., 2021).

In addition to impermeable cell barrier, there is a selective membrane-bound barrier regulated by ion channels, receptors, and transporters specifically expressed at the BBB. These molecules include ATP-binding cassette (ABC) transporters expressed in brain ECs, such as multiple drug resistance protein 1 (MDR1), permeability glycoprotein (P-gp), multiple resistance-associated protein 4 (MRP4), and breast cancer resistance protein (BCRP); these protein largely limit the permeability of the neurotherapeutic agents (e.g., anticancer drugs or kinins) through the BBB (Ueno et al., 2010; Alyautdin et al., 2014; Aday et al., 2016; Begicevic and Falasca, 2017; Gil-Martins et al., 2020). The transport of the polypeptides and proteins across the BBB requires the assistance of a series of receptor-binding molecules, such as insulin, insulin-like growth factors (IGF-I and IGF-II), angiotensin, and transferrin (Tf), which undergo receptor-mediated endocytosis (Wang et al., 2009; Soni et al., 2010). Another metabolic barrier is driven jointly by the complex and widely expressed influx/efflux transporters in the BBB. It is well known that P-glycoprotein (P-gp) and selective multidrug resistance protein-1 (MRP-1) are expressed at the high levels in the BBB and act as the efflux channels to confine the therapeutic drugs in combination with metabolic enzymes expressed by ECs (Lee et al., 2001; T. Ronaldson and P. Davis, 2012). Thus, the efficiency of the transport of most cargos from the blood circulation into the brain through the BBB is regulated by various transport systems.

Mounting evidence indicates that the permeability of the BBB is pathologically altered in several CNS diseases, such as neurodegenerative diseases, resulting in the aberrant expression of pivotal carrier/receptor-mediated transporters (e.g., P-gp and neuropeptides) and BBB efflux proteins (Zatta et al., 2009; Meairs, 2015; Logan et al., 2019; Bonsack et al., 2020). Therefore, insight into the pathophysiological characteristics of CNS barriers is essential for the development of a safe and efficient carrier system. The regulation of these transporter proteins and CNS barriers may provide new strategies for targeting of the brain by for neuroprotection and therapy.

### Blood-Cerebrospinal Fluid Barrier (BCSFB)

The BCSFB is formed by a rich vascular network surrounding choroid plexus epithelial cells, located in the choroid plexus and meninges, which secrete cerebrospinal fluid into the ventricular system. where it is secreted into the ventricular system by choroid plexus epithelial cells. Similar to many other secretory epithelial cells, but unlike the endothelial cells of the brain capillaries that form the BBB, the endothelial cells of the choroid plexus capillaries are fenestrated. The barrier is composed of choroid plexus epithelial cells and their TJs and restricts the movement of small polar molecules. Thus, the BCSFB also regulates the permeability to nutrients or xenobiotics.

Notably, similar to the BBB, the BCSFB not only acts as both a physical barriers and an enzymatic barrier via endothelial or epithelial cells. These cells express not only a series of cytoplasmic and membrane-related enzymes that can effectively metabolize biologically active drugs, but also express many transport proteins and ion channels. These characteristic enzymatic reactions and polarized expression of proteins are clearly of considerable concern for the design of drug carriers targeting the brain.

### Strategies Across the Brain Barriers

A variety of strategies have been developed to overcome the challenge of transporting the drugs across the BBB; however, the discoveries of most of strategies have not resulted in significant advances. Table 2 describes the strategies to improve the BBB penetration of active drugs/agents. New advances in nanotechnology have produced various opportunities in the field of CNS disorders because nanocarrier systems have been shown to load poorly distributed drugs in the brain, traverse the cellular/metabolic barrier regions of the BBB, and efficiently deliver the drugs into the brain parenchyma (Poovaiah et al., 2018; Prasanna and Upadhyay, 2021; Wang et al., 2021). For example, Harbi et al. designed sertraline (Ser-HCl)-loaded pegylated and glycosylated liposomes. The results of analysis of the transport in endothelial polyoma cells of the mouse brain showed that glycosylated liposomes have a greater ability to target the cerebellum than PEGylated liposomes (Harbi et al., 2016).

**TABLE 2:** Strategies for drug delivery to access the brain.

Type	Advantages	Limitation	References	
Bypass BBB				
ICV	High drug concentration, no metabolic intervention	Invasive injury, infection, elevated intracranial pressure		(Pappu et al., 2016; Heldt et al., 2019; Kazkayasi et al., 2022)
Intrathecal	Less invasive	Infection, dose-dependent drug resistance		(Falagas et al., 2007; Jain et al., 2019; Nau et al., 2021; Sari et al., 2021)
Intranasal	Rapid absorption, no first pass effect on, non-invasive	Poor bioavailability, low drug concentration		(Dowling et al., 2008; Grassin-Delyle et al., 2012)
Intraparenchymal	More clinical prospects	Invasive infection, tissue injury, obvious side effects		(McAteer and Evan, 2008; Tedford et al., 2015)
Across the BBB				
Drug lipidation	Increased BBB permeability	Increased drug efflux, nonspecific systemic administration		(Pardridge, 2003; He et al., 2018)
Prodrug	Drug solubility improvement/absorption	Poor stability, significant toxicity		Jafari et al. (2019)
Analog-based drug design	Suitable for free drug Good stability, good bioavailability, no invasive injury, small side effects	Drug molecule/capacity size is limited, CNS complications, endogenous nutrient transport interference		Baroud et al. (2021)
Nanocarriers system		Potential toxicity depends on the material used		(Bajracharya et al., 2019; de Souza et al., 2020; Wang et al., 2020)

The transport of drug nanocarrier systems across the BBB mainly involves the following mechanisms: 1) receptor-mediated endocytosis; 2) adsorptive-mediated endocytosis; 3) carrier-mediated transport; 4) passive diffusion; 5) efflux pump inhibition; and 6) the transient opening of the TJs of BBB. Surface functionalization of nanocarriers is a potential strategy to facilitate crossing of the BBB, enabling their entry into the brain via a transcellular pathway due to their specific targeting. Thus, the development of optimal drug delivery systems for CNS diseases should consider not only the ability to cross the BBB but also the ability to target and accumulate the drugs. Nanoscale particles can traverse the smallest capillaries, previous studies have demonstrated that after intravenous administration, the particles in the range of 5–10 nm are rapidly removed by the kidney, whereas the particles ranging from 10 to 50 nm are small enough to traverse the capillaries. Additionally, the particles ranging from 50 to 100 nm in size have the longest cycle lifetime, and the particles with a diameter larger than 100 nm are usually blocked by the spleen and removed by phagocytosis, resulting in a short blood circulation time (Vinogradov et al., 2002; De Jong et al., 2008; Kreyling et al., 2014).

Modifying the carrier system with targeting ligands showed better trans-BBB efficiency. The surface of polyamidoamine (PAMAM) dendrimers was conjugated to transferrin (Tf) for improved drug delivery to the brain. The results demonstrated that surface-modified dendrimers show better BBB transport ability in physiological environments.

## Emergence of the Nanogel as a Nanocarrier System

Recently developed available drug nanocarriers for the management of CNS diseases, range from more conventional formulations (e.g., liposomes, solid lipid nanoparticles, polymeric nanoparticles) to advanced formulations (e.g., nanocapsules, albumin, dendrimers (Vigani et al., 2020), and nanogels). Among them, nanogels have been shown to achieve a suitable drug pharmacokinetic profile and higher efficacy and safety compared with other drug nanocarriers.

### Properties of the Nanogel

Vinogradov et al. initially introduced the term “NanoGel” in 1999 to describe the particles of a hydrophilic polymer network (PEG-PEI) obtained by crosslinking polyethylene glycol (PEG) and polyethyleneimine (PEI) that were able to deliver antisense oligonucleotides (Zhao et al., 2015). These hydrogel nanoparticles are generally defined as three-dimensional colloidal hydrogel nanoparticles obtained by physical or chemical crosslinking of the polymers with a diameter < 200 nm, thus possessing both the advantages of a hydrogel and characteristics of a nanocarrier system (Goldberg et al., 2007; Zhang et al., 2021a). Traditionally, nanogels have been classified as physically or chemically covalently cross-linked according to the synthesis method. Nanogels can also be classified based on the network structure including hollow, core-shell, hairy, multilayered, and core-shell core cross-linking. In addition, according to their responses to environmental stimuli, they can also be divided into response and nonresponse types.

**FIGURE 3:**
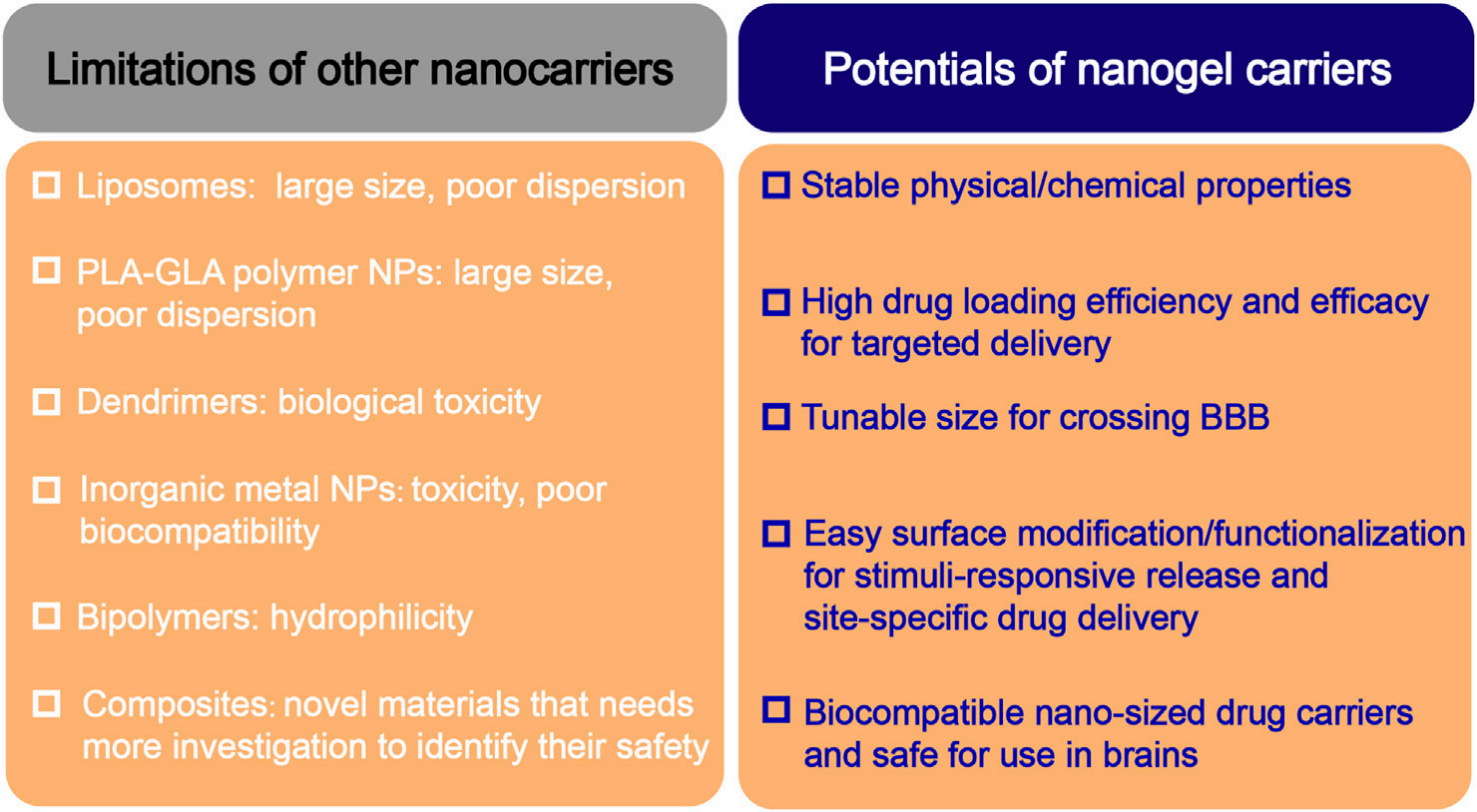
Potentials of nanogels for CNS drug delivery compared with other available nanocarriers.

Similar to the hydrogels, the hydrophilic groups in the polymeric structure of nanogels provide for a high water retention capacity (Amoli-Diva et al., 2017; Mauri et al., 2021; Stawicki et al., 2021). Nanogels have unique advantages in CNS drug delivery, especially in increasing the penetration of the drugs through the BBB, enhancing the stability of the bioactive molecules against enzymatic degradation, and reducing the cytotoxic side effects. Compared with other nanocarriers, nanogels have the following unique characteristics (Figure 3):1) Tunable nanosize: nanogels have a large specific surface area and, more importantly, can be engineered with an adapted nanosize based on the target tissue/organ, enabling them to efficiently cross cellular and biological barriers. 2) Colloidal stability: nanogels possess higher stability in physiological environments. 3) Swelling behavior: swelling/deswelling is one of the most important properties of nanogels and can be controlled by their design with suitable parameters (such as polymers, cross-linking forms, and functional structures). Furthermore, these behavior properties can be altered by responding to external stimuli. 4) Drug loading and easy surface modification: the diversity of polymeric materials and simple modification of their physical or chemical characteristics enables the creation of nanogels with versatile formulations. Depending on the crosslinked polymer network, such as the hydrophilic/lipophilic groups of the monomers, surfactants, surface charge, and crosslinking agents, various types of nanogels can deliver almost all types of therapeutic agents, including active biomacromolecules (DNA and SiRNA), hydrophobic/hydrophilic drugs, proteins, vaccines and even immunetherapeutics (Coviello et al., 2007; Lombardo et al., 2020; Chander et al., 2021; Tang et al., 2022). 5) Active targeting and controlled release: nanogels can be synthesized by crosslinking natural (e.g., alginate, dextran, and hyaluronic acid) or artificial polymers (e.g., methylcellulose, chitosan, and cyclodextrin). These polymers are nontoxic and stable and ensure high cell viability in the studies, demonstrating that nanogels are inherently biocompatible and biodegradable, which can avoid excessive accumulation in the tissues (Soni et al., 2016b; Mathew et al., 2018). 6) Non immune response: due to their high water-holding capacity a which enables them to absorb large amounts of nonimmunoreactive liquids, usually nanogel formulations do not produce any immune response; 7) Biodegradability: nanogels are synthesized from natural materials or polymers, which can be degraded in a nontoxic manner in living organisms and thus avoid organ accumulation.

### Progress of Nanogel Drug Carrier Systems

**FIGURE 4:**
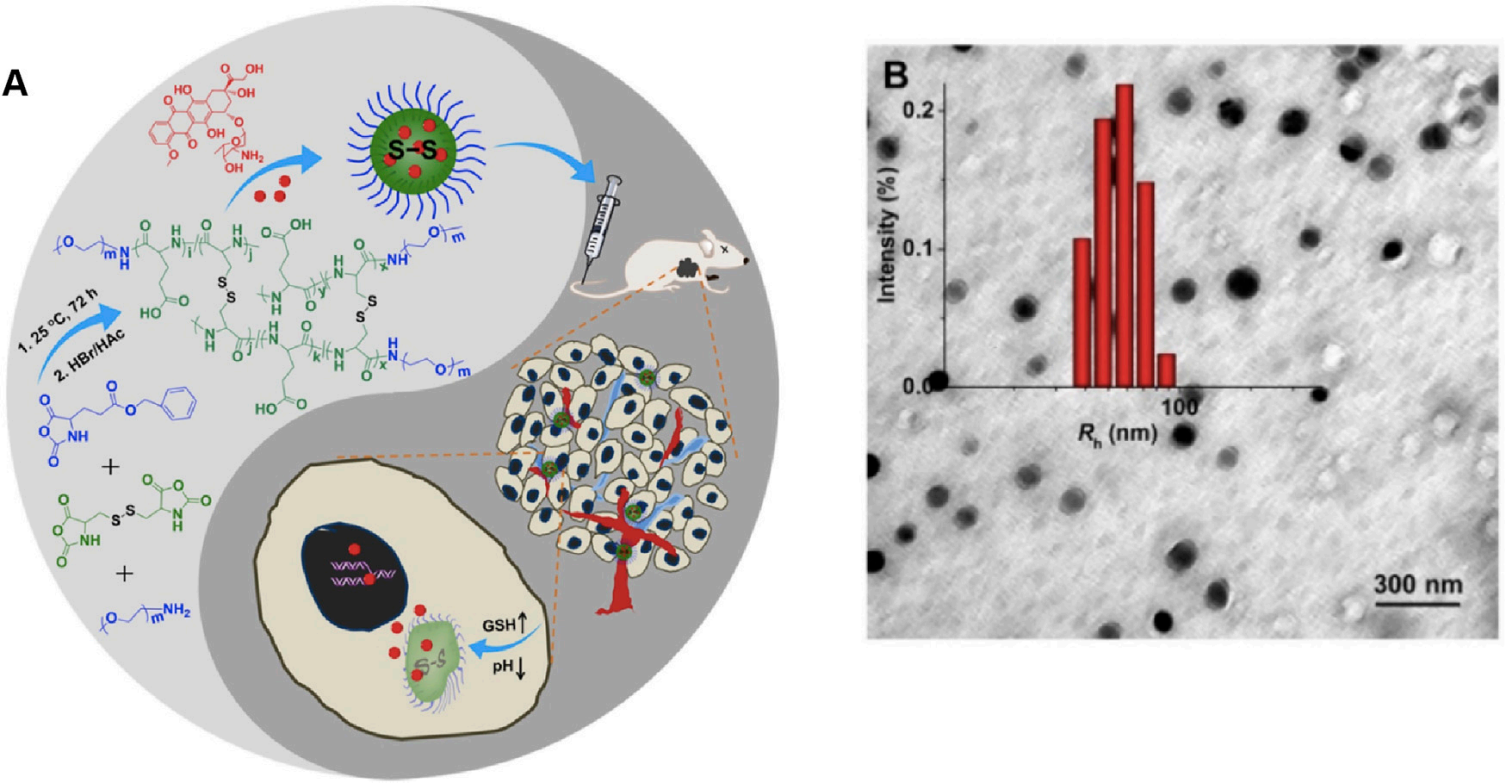
**(A)** Synthetic pathway for mPEG−P (LG-co-LC) nanogel, illustration of DOX encapsulation by nanogel, and its circulation, intratumoral accumulation, endocytosis, and targeted intracellular DOX release after intravenous injection (Huang et al., 2015). **(B)** Typical TEM micrographs and Rh NG/DOX. Copyright 2017 Ivyspring International Publisher.

Nanogels can be accurately modified with respect to their shape, charge, and surface function in response to various internal stimuli (changes in pH, redox conditions, or enzymes), which are usually associated with the majority of physiological conditions *in vivo* because most pathological processes generally induce certain changes in pH, redox levels, or specific complementary ligand expression levels. A continuous increase in the availability of the functional and macromolecular monomers can expand the response range of these nanogels (Stuart et al., 2010; Sun et al., 2014). Furthermore, comparison with traditional nanomaterial-based controlled release systems indicates that stimulus-responsive nanogels can react to external stimuli, such as light, electricity, and magnetism, which can control drug release by reversible expansion or contraction of the gels (Zhang et al., 2020). Zhang et al. formed ultra-pH-sensitive nanogels through the self-assembly of an ultra-pH-sensitive hydrogel from the chiral peptide derivative ferrocene-diphenylalanine (FC-FF). The material precisely responds to the changes in pH over a very narrow range (pH 5.7–5.9) (Liu et al., 2020a). Polypeptide, a promising biomedical polymer with biodegradability and biocompatibility, was approved in 1906 α- Ring opening polymerization (ROP) of amino acid n-carboxylic anhydride (NCA) was synthesized for the first time. At present, peptide based nano gel has been applied to the targeted delivery of therapeutic drugs for various diseases (Shi et al., 2017). Previously, our team synthesized the dual-response nanogels, NG/DOX, which respond simultaneously to a low pH level and a high GSH level. After intravenous injection of NG/DOX into tumor-bearing mice, the drug release from nanogel is triggered at a low pH level and a high GSH level. The antitumor effect of NG/DOX is far superior to that of free DOX hydrochloride, and nanogel has extremely low cytotoxicity (Figure 4).. (Huang et al., 2015; Suhail et al., 2019)

**FIGURE 5:**
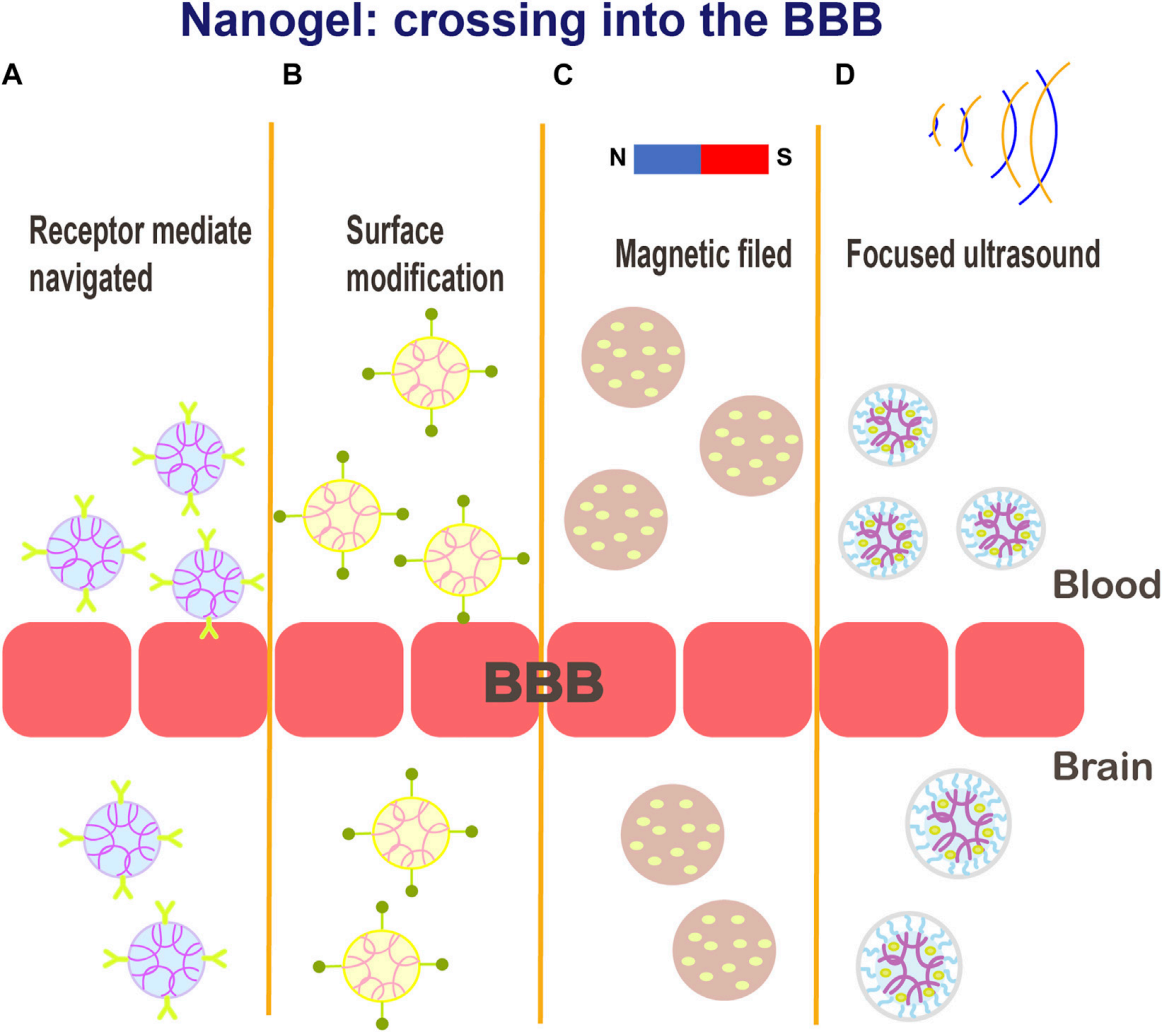
Schematic illustration of the various potential mechanisms for crossing the BBB: **(A)** receptor-mediated endocytosis; **(B)** functionalized ligands that recognize cognate receptors on target organs or tissues; **(C–D)** magnetic field or ultrasound mediated delivery.

Ion-induced gelation has attracted considerable attention due to environmentally friendly and time-controlled properties (Lee et al., 2021). Recently, researchers have developed stimuli-responsive DNA noncationic nanogels that can be used for targeted delivery of combination cancer therapeutics with high biocompatibility (Oh et al., 2007). Degradable nanogels loaded with rhodamine B isothiocyanate dextran (RITC DX) were shown to be degraded into a polymeric sol in a reducing environment, thus releasing the encapsulated carbohydrate drugs (Vinogradov, 2010). Based on nanogel technology, many therapeutic strategies for the delivery/release of therapeutic drugs to the brain have been explored to achieve active targeting: 1) by attaching functionalized ligands that recognize homologous receptors on the target organs or tissues, 2) due to noninvasive responsiveness to magnetic field or ultrasound to disrupt the BBB, and 3) due to shutter peptide-mediated BBB crossing (Figure 5). (Ganguly et al., 2014; Chaurasiya et al., 2016; Wei et al., 2021) Furthermore, nanogels with encapsulated drugs can be delivered by a variety of methods, such as intravenous or intraperitoneal injection, oral administration, and nasal and intraocular drug delivery (Verma et al., 2011; Zhao et al., 2021). Reports have suggested that PEGylation renders nanogel surface more hydrophilic, shields the drugs, and provides steric hindrance to avoid the interactions with serum proteins, endowing nanogels with a “stealth” feature (Kamaly et al., 2012; Sun et al., 2014).

The modulation of nanogels (e.g., ligands complementary to receptors) can direct them to the affected tissues with lesions, which differentially express the corresponding receptors, thereby facilitating the uptake and retention of the drugs at a target site (Saha et al., 2021). Vinogradov et al. synthesized a new system based on a nanogel network of crosslinked PEG and PEI, which is capable of efficiently delivery of ODNs to the brain across the BBB. The transport efficiency was further improved when the surface of nanogels was modified with Tf or insulin. The results of distribution studies in a mouse model showed that the accumulation of ODN in the brain was increased by more than 15-fold after 1 h of intravenous injection compared with the accumulation of unincorporated ODN, and the accumulation of free ODN in the liver and spleen was reduced by 2-fold, implying that clearance of ODN from the blood was not accelerated (Vinogradov et al., 2004). Zwitterionic-based nanogels have greatly broadened the applications of nanogels in drug delivery by virtue of their characteristics such as superhydrophilicity (Peng et al., 2020). In this context, zwitterionic polysulfamide nanogels (PMEDAPA) modified with transferrin (Tf) were synthesized as drug carriers that effectively respond to hyperthermia. These nanogels have their tumor-targeting features shielded at normal temperature; at high temperature, tumor targeting is achieved to enhance the accumulation of chemotherapeutic drugs in the tumor. These finding provides an exciting rationale to achieve tumor targeting of nanogels and to enable on-demand drug release in microwave heating-assisted cancer therapy in a clinical setting (Muresanu et al., 2019).

Overall, nanogels are more novel and advanced nanocarrier that are characterized by superior efficacy, bioavailability, and favorable drug pharmacokinetics, render their the potential side effects are greatly reduced.

## Applications of the Nanogels for CNS Drug Delivery

The advantages of simple and stable synthetic routes, controllable drug release, and high targeting efficiency make nanogel drug carriers one of the preferred options for the treatment of various CNS diseases, such as stroke, neurodegenerative disorders, epilepsy, traumatic brain injury, and brain tumors. Table 3 summarizes several major studies related to the design of nanogels used for CNS drug delivery.

**TABLE 3:** Summary of representative studies on nanogels for CNS drug delivery.

Formulations	Function	Drugs/Agents	Outcomes	Mechanism Across BBB	References
Nanogel/ODN	Transferrin/insulin-targeting	ODN	Nano-ODN accumulation increased 15 fold in the brain, whereas free ODN accumulated in a large amount in liver and spleen	Ligand- mediated	Vinogradov et al. (2004)
PEG-PEI	Controlled release	Antisense oligonucleotide	Increased *in vitro* uptake, controlled and sustained oligonucleotide release, higher uptake in the brain tissue	Charge adsorption-mediated	Wong et al. (2012)
HCFU	Sustained release	5-fluorouracil	Nanogel coating with polysorbate increased the accumulation from 0.18 to 0.52%, largely enhanced uptake in the brain tissue	Carrier-mediated	Xu et al. (2019)
Dex-FFFKE-ss-EE	Redox-responsive	Taxol/HCPT	Co-delivery nanogel system cantained two complementary anti-cancer drugs, extended drug realse and improved the stability of drugs.	Endocytosis	Teng et al. (2018)
Hollow nanogels (nUK)	Ultrasound-responsive	uPA	nUK has controlled released of uPA taget the clot site under ultrasound, not only enhanced the circulation of the drug, but also increased the safety.	Ultrasound stimulation	Xu et al. (2019)
PCgels	Immunotherapy	T lymphocyte	PCgels had suitable pore size to possess the cellular compatible with T lymphocytes, which retained their localized anti-glioblastoma activity in the PCgels	T lymphocyte-mediated	Mao et al. (2012)
Nano-NRTIs	Anti-viral	NRTIs	Nano-NRTIs exhibited high efficacy against HIV-1 in macrophages at a drug level as low as 1 μmol/L and decreased cytotoxicity compared to NRTIs	Macrophage phagocytosis	Vinogradov et al. (2010)
PEG-PVA/micelle	pH/redox-responsive	TMD/CF	Dual drugs of PEG-PVA/micelle were released significantly increased in alkaline environment	PH stimulation	Mao et al. (2012)

### Ischemic Stroke

Ischemic stroke occurs when a blood clot or embolus locally blocks the middle brain artery, which accounts for 85% of all types of stroke (Houng et al., 2014). Currently, the thrombus-dissolving agents, such as tissue plasminogen activator (tPA), are available for the treatment of ischemic stroke; however, a narrow time window for the use of these agents and cerebral ischemia/reperfusion injury often cause serious pathological reactions, which produce unsatisfactory results of these conventional treatment approached for ischemic stroke (Mihalko et al., 2022). Nanogels are expected to expand the arsenal of ischemic stroke treatment strategies by achieving brain targeting of the drugs and local controlled release.

Mihalko et al. designed a fibrin-specific nanogel (FSN) that can be used for the targeted delivery of tPA. *In vivo* experiments confirmed that tPA-FSNs can modulate fibrin/fibrinogen and platelets in thrombi. The distribution of both FSN and tPA-FSNs showed potential clearance and very low toxicity after 24 h. (Cui et al., 2016) A recent study suggested that a new form of urokinase (United Kingdom)-containing PEG-conjugated nanogel with pH-sensitive properties (PEG-UK) was designed to release the payload at a certain pH value. PEG-UKs were detected at the regions of microcirculation with low pH in a rat model of ischemic stroke. Wei Cui at el. demonstrated that the administration of PEG-UKs reduces the infarct volume of ischemic stroke, and this effect protects the BBB, inhibits apoptosis, and decreases neurotoxicity (Kleindorfer et al., 2005). However, high incidence of hemorrhagic events and failure to hospitalize in time usually limit the application of thrombolytic drugs. Statistical data show that very few patients receive thrombolytic therapy within 3 h after ischemic stroke (He et al., 2021). Multiple findings have shown that the generation of reactive oxygen species (ROS) or reactive nitrogen species (RONS) associated with cerebral ischemia and reperfusion worsen the conditions in patients. To reduce ischemic injury caused by oxidative stress, Zhang et al. developed artificial nanogel-zymes with multiple enzyme activities, which were able to provide neuroprotection against ischemic stroke by scavenging RONS. In a rat model of ischemic stroke, RONS levels were significantly reduced, and side effects were minimal (Liu et al., 2021).

The latest studies have shown that administration of microRNAs (miRNAs) can promote blood vessel growth and help restore the function of damaged tissues. Liu et al. encapsulated miRNAs in a nanocapsule platform, which systematically and effectively delivered miRNAs (Davis, 2016). However, the delivery of miRNA by nanogel delivery platform against cerebral ischemia has yet to be investigated. Moreover, future work should assess in detail the ability of these nanoformulations to be translated to the clinic.

### Brain Tumors

Specific targeted drug delivery is the future of brain tumor therapy. Gliomas are the most prevalent and malignant CNS tumors, with a median patient survival of less than 15 months despite aggressive use of surgery combined with radiotherapy and chemotherapy (Kim et al., 2018). The blood-brain tumor barrier (BBTB) forms with tumor progression, which blocks almost all small-molecule chemotherapeutics and biomacromolecules. In addition to the limitations of the barriers, most therapeutic agents for glioma are the substrates of biological barrier efflux transporters, and a combination of these obstacles contribute to a high rate of treatment failure (Agarwal et al., 2011; Feng et al., 2019). Gliomas, similar to other solid tumors, have a special tumor microenvironment (TME) characterized by hypoxia, low pH, and chronic inflammation. Intriguingly, the extracellular glutathione concentrations of gliomas are up to 1,000-fold higher than the extracellular glutathione concentrations in the normal tissues, and the pH values in the vicinity of tumor cells are significantly lower (Marí et al., 2009; Wen et al., 2019).

Based on specific characteristics of the tumor tissue, various stimulus-responsive nanogel drug delivery networks have been developed for antitumor drug delivery. Methotrexate, doxorubicin, rituximab, and temozolomide are routine chemotherapeutic agents toxic for glioma cells, which have been formulated as nanomedicines to significantly improve the efficiency of the BBB crossing. The membrane protein connexin 43 (Cx43) and brain-specific anion transporter (BSAT1) are characteristically expressed in gliomas and the adjacent tissues. Recently, cisplatin was loaded into nanogels conjugated to Cx43 and BSAT1 monoclonal antibodies. MRI analysis of tumor-bearing rats showed that the nanoformulation achieved targeted antitumor effects (Gadhave et al., 2021). A recent study demonstrated successful loading of teriflunomide into a nanolipid-based (NLC) carbopol-gellan gum nanogel (TNLCGHG) for the treatment of brain glioma via intranasal administration. These gels were demonstrated to prolong blood circulation and showed significant tumor-suppressive effects (Zhang et al., 2021b). Nanogel formulations have been developed as promising contrast agents. Jiang et al. developed Cy5.5-Lf-MPNA nanogels by labeling lactoferrin (Lf) with Cy5.5. This preparation can target tumor tissue and achieve MR/fluorescence imaging with high sensitivity and specificity in the acidic environment of glioma tissue (Cui et al., 2016). Similarly, Lf/phenylboronic acid (PBA)-reduction-sensitive dual-target nanogels (Lf-DOX/PBNG) were developed for the delivery to deliver doxorubicin (DOX) for glioma therapy. The effective accumulation of Lf-DOX/PBNG was 12.37 times greater than that of free DOX solution (Rizzi et al., 2014).

### Alzheimer’s Disease

Alzheimer’s disease (AD) is the most common cause of dementia, with more than 50 million people worldwide currently living with dementia (DeTure and Dickson, 2019). Clinical therapeutic approaches for AD focus on lowering the levels of the toxic forms of the amyloid beta (Aβ) peptide and τ protein to effectively delay the progression of the disease (Giacobini and Gold, 2013; Yang et al., 2021). A range of limitations account for poor efficacy of most therapeutic drugs, such as hydrophobicity, poor BBB permeability, rapid metabolism, and strong tissue toxicity. Various nanogel technologies have enabled the treatment of neurodegenerative diseases (Jiang et al., 2018).

The development of the dual inhibitor nanosystems is expected to effectively target the inhibition of Aβ aggregation and cytotoxicity induced by drug delivery systems. The biocompatible nanogels of cholesterol-bearing pullulan (CHP) were shown to be able to significantly inhibit the formation of Aβ fibrils. Epigallocatechin-3-gallate (EGCG) and curcumin are able to effectively inhibit Aβ aggregation. These two inhibitors were linked via a modification of hyaluronic acid (HA). The results showed that the EGCG and curcumin dual-modified nanogels (CEHA), which were synthesized by self-assembly, induced 69 and 55% higher inhibition than EGCG- or EHA-loaded single-modified nanogels, respectively. The results of an *in vitro* toxicity assay showed that CEHA significantly improved the viability of SH-SY5Y cells (Zhang et al., 2021c). Another approach to AD therapy involves scavenging of excess ROS induced by mitochondrial dysfunction and inflammatory factor that are overactivated in the brain (McNaught et al., 2002). Oxytocin-loaded angiopep-2-modified AOC NGs were developed to cross the BBB via the transcytosis of a surface-loaded ligand (ANG) for enrichment in the AD lesions. AOC NGs can block the ERK/p38 MAPK and COX-2/iNOS NF-κB signaling pathways, showing the ability to effectively inhibit microglial activation and reduce inflammatory cytokine levels.

Advances in nanotechnology have enabled new prospects for clinical therapeutic strategies for AD; however, the field of AD nanotherapeutics faces several challenges. AD may lead to a variety of clinical complications; hence, the design of the nanogel drugs with multiple drug candidates to achieve a synergistic effect may enhance the benefits of AD treatment.

### Parkinson’s Disease

Parkinson’s disease (PD) is the second most common neurodegenerative disorder characterized by the death of dopaminergic neurons in the substantia nigra and the formation of Lewy bodies (Hawthorne et al., 2016). Dopamine therapy is the primary treatment option for PD; however, the disease is currently considered incurable. The demands for nanomedical studies of PD are mainly focused on achieving a stable concentration of dopamine in the brain (Jiang et al., 2018; Khan et al., 2018).

PEGylated nanogels were engineered to load dopamine and modified ligands of the transferrin receptor. The results of *in vivo* experiments demonstrated that the concentration of dopamine in the brain obtained using this nanogel was nine times higher than that obtained in a rat model treated with free dopamine. The removal of Lewy bodies is another effective strategy for the treatment of PD (Liu et al., 2020b). A study developed the Lewy body antagonist NanoCA using a self-assembly reaction. NanoCA targets the brain, releases its cargo in a controlled manner, and protects the neurons from the neurotoxicity of PD inducers in the animal models. In PD animal models, the nanomaterials are administered by local injection or transdermal absorption. Ropinirole nanogels were proved to enhance the efficacy of transdermal absorption, thus increasing the bioavailability up to two-fold compared with transdermal methods of delivery of the unincorporated free drug (Jafarieh et al., 2015). Jafarieh et al. prepared ropinirole hydrochloride (RH)-loaded chitosan nanoparticles (RH-CSNPs) by the ionic gel method. RH-CSNPs with nasal mucosal absorbability continuously released their cargo for 18 h, and the RH concentration in the brain was significantly increased after intranasal administration (Leoni and Caccia, 2014). The first-line treatment for patients with Parkinson’s disease is natural oral levodopa, which has shown higher efficiency than synthetic levodopa. C Chittasupho et al. encapsulated native levodopa from *M. pruriens* seed extract into nanogels for incorporated into a jelly as a functional food for patients with Parkinson’s disease.

Finally, although the prospects of using nanogel technology to treat neurodegenerative diseases are very attractive, the actual studies performed to date remain only experimental.

### Huntington’s Disease

Huntington’s disease (HD) is an autosomal dominant neurodegenerative disorder (Arrasate and Finkbeiner, 2012). The known molecular mechanism of HD involves a single mutation of the huntingtin (HTT) gene exon 1, which leads to polyQ expansion, resulting in the misfolding and aggregation of the huntingtin protein in the brain (Li et al., 2020). However, the molecular mechanism by which HTT mutation causes neuronal death remains unclear. At present, many studies have demonstrated that short interfering RNAs (siRNAs) can silence the expression of the mutant proteins, and this application is one of the most recent and promising therapeutic strategies (Godinho et al., 2013).

Godinho et al. developed modified amphiphilic β-cyclodextrin (CD) oligosaccharide molecules as novel neuronal siRNA vectors. The results showed that CD nanoparticles are stable in artificial cerebrospinal fluid. The nanocarrier complex reduces HTT gene expression in ST14A-Htt120q rat striatum cells and primary human HD fibroblasts. A single injection of the CD-siRNA nanoparticles significantly reduces HTT expression in the striatum in a mouse HD model, and multiple injections can alleviate the motor dysfunction in HD mice. In addition, low toxicity of CD-siRNA nanoparticles has been observed in vitro toxicity experiments (Löscher et al., 2020). Numerous efforts have demonstrated the potential efficacy of the nanogel drug delivery systems; however, limited data on *in vivo* toxicity suggest a need to determine potential long-term systemic toxicity.

### Epilepsy

Epilepsy is the second most frequent chronic disease of the CNS. Approximately 30% of patients with epilepsy are characterized by poorly controlled drug release and drug resistance during clinical treatment (Hanada, 2014; Shringarpure et al., 2021). Nanogel technology promises to revolutionize the treatment strategies for epilepsy using unique advantages due to the ability to cross the BBB without toxic effects to the brain and other tissues.

Lamotrigine is a broad-spectrum antiepileptic. Only a small amount of potent lamotrigine can exert its antiepileptic effects through the BBB after oral absorption (Xu et al., 2022). The group of Xu designed a polymer hydrogel that specifically responded to electromagnetic radiation. The results of intravital imaging of rats showed that fluorescently labeled lamotrigine nanogels are enriched in the rat brain 3 h after intravenous injection, indicating that lamotrigine nanogels are characterized by enhanced BBB penetration. Comparison with free lamotrigine indicated that the seizures of rats in the lamotrigine nanogel group were continuously and significantly decreased (Wang et al., 2016).

Recently, Wang et al. designed electroresponsive hydrogel nanoparticles (eRHNPs) modified with brain-targeting angiopep-2 (ANG) to facilitate the delivery of the antiepileptic drug phenytoin (PHT) and subsequently developed PHT-loaded ANG-eRHNPs. These formulations achieved a high distribution in the brain and an electrical response in a rat epilepsy model, resulting in a strong release of PHT from nanogels during seizures and achieving better antiepileptic effects (Ying et al., 2014). Brain-targeted ANG peptide-modified electro-responsive nanogels have been confirmed to achieve high-efficiency BBB penetration and enhance the efficacy of alleviating epilepsy (Wilson et al., 2017).

These studies will enable the generation of safe and effective seizure therapeutics. Future attention should also be paid to human clinical trials to evaluate and select precise nanodosage forms and concentrations for subsequent clinical applications.

### Traumatic Brain Injury

Traumatic brain injury (TBI) is the main cause of death and disability in young individuals under 45 years of age and a known risk factor for chronic neurodegenerative diseases, such as AD and PD. To date, effective clinical treatments for TBI are lacking (McKee and Robinson, 2014; Gong et al., 2022).

The application of the bioactive scaffold materials is a promising method for tissue regeneration and repair (Marçal et al., 2012). Bladder stroma extracted from the porcine bladder tissue (UBM) showed good performance in promoting and supporting neural cell growth in vitro experiments (Zhang et al., 2013). To improve the biocompatibility of UBM in the brain and the effect of UBM on the function after TBI, a hydrogel-based form of UBM was developed. The results showed that the UBM-hydrogel had weak toxic effects on the healthy brain. After TBI, the application of the nanoscale UBM-hydrogel reduced the volume of the lesions and alleviated myelin sheath breakage induced by traumatic injury (Xu et al., 2019). Unexpectedly, the treatment of TBI using the UBM-hydrogel significantly improved neurobehavioral and vestibulomotor functions. The application of nanogels not only greatly improved the biocompatibility of UBM but also revealed a protective effect on the injured brain tissue. These observations provide valuable insight into the potential efficacy of nanogels in structurally damaged brain tissue.

Recently, Xu et al. successfully demonstrated that nanocapsules loaded with nerve growth factor (NGF) enabled nerve recovery and tissue remodeling in mice with spinal cord injury (Teng et al., 2018). It is suggested that nanogel technology may provide a better approach for TBI and CNS tissue regeneration engineering, which needs to be more explored in the future.

## Potential Neurotoxicity

The CNS strongly protects itself from any xenobiotics (pathogens, toxins, and foreign bodies) through strict barrier structures (Nance et al., 2014). Nanocarriers can effectively circumvent the BBB and deliver unevenly distributed drugs to the brain parenchyma (Xiao et al., 2015); however, this delivery also leads to overexposure to the nanomaterials, making the application of the nanomaterials for CNS therapy regarded as a dual-edged sword. Therefore, it is crucial to systematically investigate the potential toxicity of the nanomaterials, which is one of the biggest challenges for the clinical translation of the nanodrug delivery systems.

Existing neurotoxicity studies have shown that the nanocarriers have neurotoxic effects both *in vitro* and *in vivo* induced by the nanoparticles composed mostly of inorganic materials, whereas complete assessment of potential toxicity of nanogels in humans has not been reported (Wong et al., 2012). The present review analyzed the main reasons associated with neurotoxicity of nanogels. Chemically crosslinked nanogels may release toxic monomers from the matrix during their expected chemical degradation (Lee et al., 2021). Inflammation is one of the main mechanisms of neurotoxicity caused by the nanomaterials. Inflammation is known to eliminate foreign substances from the body and is a protective response; however, overactivation of the inflammatory response will induce substantial damage. Exposure of the brain to nanogels may stimulate glial cells, causing a strong inflammatory response of neuronal mitochondria or other organelles (Trompetero et al., 2018; Wu and Tang, 2018). The mechanism of brain inflammation induced by nanogels needs additional research, and only a few relevant studies of this subject have been performed.

In addition, neuronal exposure to the nanomaterials may induce neurodegenerative diseases. The accumulation of some fibrin aggregates or misfolded proteins is the pathological mechanism of some neurodegenerative diseases (AD, PD, and HD). Plausible interactions between the CNS and nanomaterials may exacerbate the accumulation or misfolding of these protein aggregates (Pichla et al., 2020). The results reported by Alvarez et al. suggest that the induction of a neurodegenerative disease may depend on the size and concentration of the nanoparticles and on their biocompatibility (Oberdörster, 2010). Current data of the assessments of the toxicity of the nanomaterials suggest that rigorous *in vitro*, *in vivo*, and clinical toxicity studies are needed before the clinical transformation of the nanomaterials can be achieved (Wang et al., 2022). Emphasis on detailed toxicity studies of nanogels is needed to provide evolutionary insight into the risks associated with efficient applications of these promising targeting approaches.

## Discussion

Overall, nanogel technology can provide the possibilities for advanced treatment in the field of CNS diseases (Sarkar et al., 2017). The BBB is the major anatomical and physiological dynamic barrier, representing one of the narrowest bottlenecks for successful treatment of CNS diseases; hence, the development of safer and more effective targeted nanomedicines has become a major research direction for numerous nanoformulation studies. Continuous development of new nanogel technologies, such as surface modification of the receptors/ligands and magnetic structures, enables various nanogel-based approaches to overcome the BBB to achieve targeted drug delivery. Nanogel-based technologies have been implicated in the development of the nanocarriers of various neuroprotective drugs, including nucleic acids, small-molecule peptides, nerve growth factors, and free radical scavengers (e.g., edaravone). However, the nanogel drug delivery platforms are still in their infancy (Jogani et al., 2008; Kumar et al., 2017; Patel et al., 2021). Therefore, additional in-depth studies on nanogels are required to address several issues before these agents can be widely used in the clinic.

Specific targeted delivery is a future research direction for nanogel studies. Recent discoveries of several specific peptides or receptor-targeting drugs, such as Tf, ANG and human insulin receptor, can facilitate the development of effective nanogels for brain-targeted drug delivery. However, these receptors are not specific; thus, organ/tissue specificity should also be considered in the future studies to provide more specific and rational therapeutic strategies. Due to the outstanding properties associated with the suppression of reactive oxygen species and protein misaggregation, the application of nanogels have been eagerly explored for the treatment of neurological diseases, such as ischemic stroke, AD, PD, brain tumors, and epilepsy (Liu et al., 2018). However, the efficacy of these carrier systems is hindered by the involvement of various pharmacological factors, such as drug loading, controlled release, safety, and biocompatibility *in vivo*. Furthermore, considering the complex structure and unique microenvironment of the brain, additional polymeric materials should be developed to enhance biodegradability and biocompatibility by preparing nanogels with completely eliminated possibilities of potential toxicity.

Most CNS diseases require long-term drug therapy; thus, toxicological studies of the organs, including the kidney, liver, and spleen, should be performed using nanogel formulations (Picone et al., 2018). In the future, advanced diagnostic techniques, such as magnetic resonance imaging, positron emission tomography, and computed tomography, aphy, will be needed to assess nanocarrier-related CNS toxicity.

## References

[B1] Aday S. Cecchelli R. Hallier-Vanuxeem D. Dehouck M. P. Ferreira L. (2016). Stem Cell-Based Human Blood-Brain Barrier Models for Drug Discovery and Delivery. Trends Biotechnol. 34 (5), 382–393. 10.1016/j.tibtech.2016.01.001 26838094

[B2] Agarwal S. Sane R. Oberoi R. Ohlfest J. R. Elmquist W. F. (2011). Delivery of Molecularly Targeted Therapy to Malignant Glioma, a Disease of the Whole Brain. Expert Rev. Mol. Med. 13, e17. 10.1017/s1462399411001888 21676290 PMC5048912

[B3] Ali E. S. Sharker S. M. Islam M. T. Khan I. N. Shaw S. Rahman M. A. (2021). Targeting Cancer Cells with Nanotherapeutics and Nanodiagnostics: Current Status and Future Perspectives. Seminars Cancer Biol. 69, 52–68. 10.1016/j.semcancer.2020.01.011 32014609

[B4] Alyautdin R. Khalin I. Nafeeza M. I. Haron M. H. Kuznetsov D. (2014). Nanoscale Drug Delivery Systems and the Blood-Brain Barrier. Int. J. Nanomedicine 9, 795–811. 10.2147/IJN.S52236 24550672 PMC3926460

[B5] Amoli-Diva M. Sadighi-Bonabi R. Pourghazi K. (2017). Switchable On/off Drug Release from Gold Nanoparticles-Grafted Dual Light- and Temperature-Responsive Hydrogel for Controlled Drug Delivery. Mater. Sci. Eng. C 76, 242–248. 10.1016/j.msec.2017.03.038 28482523

[B6] Arrasate M. Finkbeiner S. (2012). Protein Aggregates in Huntington's Disease. Exp. Neurol. 238 (1), 1–11. 10.1016/j.expneurol.2011.12.013 22200539 PMC3909772

[B7] Bae K. H. Lee J. Y. Lee S. H. Park T. G. Nam Y. S. (2013). Optically Traceable Solid Lipid Nanoparticles Loaded with siRNA and Paclitaxel for Synergistic Chemotherapy with *In Situ* Imaging. Adv. Healthc. Mater. 2 (4), 576–584. 10.1002/adhm.201200338 23184673

[B8] Baeten K. M. Akassoglou K. (2011). Extracellular Matrix and Matrix Receptors in Blood-Brain Barrier Formation and Stroke. Devel Neurobio 71 (11), 1018–1039. 10.1002/dneu.20954 PMC3482610 21780303

[B9] Bajracharya R. Song J. G. Back S. Y. Han H.-K. (2019). Recent Advancements in Non-invasive Formulations for Protein Drug Delivery. Comput. Struct. Biotechnol. J. 17, 1290–1308. 10.1016/j.csbj.2019.09.004 31921395 PMC6944732

[B11] Baroud M. Lepeltier E. Thepot S. El-Makhour Y. Duval O. (2021). The Evolution of Nucleosidic Analogues: Self-Assembly of Prodrugs into Nanoparticles for Cancer Drug Delivery. Nanoscale Adv. 3 (8), 2157–2179. 10.1039/d0na01084g PMC9418958 36133769

[B12] Battaglia L. Gallarate M. Peira E. Chirio D. Muntoni E. Biasibetti E. (2014). Solid Lipid Nanoparticles for Potential Doxorubicin Delivery in Glioblastoma Treatment: Preliminary *In Vitro* Studies. J. Pharm. Sci. 103 (7), 2157–2165. 10.1002/jps.24002 24824141

[B13] Begicevic R. R. Falasca M. (2017). ABC Transporters in Cancer Stem Cells: Beyond Chemoresistance. Int. J. Mol. Sci. 18 (11), 2362. 10.3390/ijms18112362 PMC5713331 29117122

[B14] Bhia M. Motallebi M. Abadi B. Zarepour A. Pereira-Silva M. Saremnejad F. (2021). Naringenin Nano-Delivery Systems and Their Therapeutic Applications. Pharmaceutics 13 (2), 291. 10.3390/pharmaceutics13020291 33672366 PMC7926828

[B15] Bonsack B. Corey S. Shear A. Heyck M. Cozene B. Sadanandan N. (2020). Mesenchymal Stem Cell Therapy Alleviates the Neuroinflammation Associated with Acquired Brain Injury. CNS Neurosci. Ther. 26 (6), 603–615. 10.1111/cns.13378 32356605 PMC7248547

[B16] Buyukkoroglu G. Senel B. Basaran E. Yenilmez E. Yazan Y. (2016). Preparation and *In Vitro* Evaluation of Vaginal Formulations Including siRNA and Paclitaxel-Loaded SLNs for Cervical Cancer. Eur. J. Pharm. Biopharm. 109, 174–183. 27793757 10.1016/j.ejpb.2016.10.017

[B17] Chander S. Kulkarni G. T. Dhiman N. Kharkwal H. (2021). Protein-Based Nanohydrogels for Bioactive Delivery. Front. Chem. 9, 573748. 10.3389/fchem.2021.573748 34307293 PMC8299995

[B18] Chaurasiya B. Mahanty A. Roy D. Shen Y. Tu J. Sun C. (2016). Influence of Tumor Microenvironment on the Distribution and Elimination of Nano-Formulations. Cdm 17 (8), 783–798. 10.2174/1389200217666160607093347 27280439

[B19] Chen Z. Lv Z. Zhang Z. Weitz D. A. Zhang H. Zhang Y. (2021). Advanced Microfluidic Devices for Fabricating Multi‐structural Hydrogel Microsphere. Exploration 1 (3), 1. 10.1002/exp.20210036 PMC10191056 37323691

[B20] Cojocaru F. D. Botezat D. Gardikiotis I. Uritu C. M. Dodi G. Trandafir L. (2020). Nanomaterials Designed for Antiviral Drug Delivery Transport across Biological Barriers. Pharmaceutics 12 (2), 171. 10.3390/pharmaceutics12020171 PMC7076512 32085535

[B21] Coviello T. Matricardi P. Marianecci C. Alhaique F. (2007). Polysaccharide Hydrogels for Modified Release Formulations. J. Control. Release 119 (1), 5–24. 10.1016/j.jconrel.2007.01.004 17382422

[B22] Cui W. Liu R. Jin H. Lv P. Sun Y. Men X. (2016). pH Gradient Difference Around Ischemic Brain Tissue Can Serve as a Trigger for Delivering Polyethylene Glycol-Conjugated Urokinase Nanogels. J. Control. Release 225, 53–63. 10.1016/j.jconrel.2016.01.028 26795685

[B23] Date A. Joshi M. Patravale V. (2007). Parasitic Diseases: Liposomes and Polymeric Nanoparticles versus Lipid Nanoparticles☆. Adv. Drug Deliv. Rev. 59 (6), 505–521. 10.1016/j.addr.2007.04.009 17574295

[B24] Davis M. E. (2016). Glioblastoma: Overview of Disease and Treatment. Clin. J. Oncol. Nurs. 20 (5 Suppl. l), S2–S8. 10.1188/16.CJON.S1.2-8 PMC5123811 27668386

[B25] De Jong W. H. Hagens W. I. Krystek P. Burger M. C. Sips A. J. A. M. Geertsma R. E. (2008). Particle Size-dependent Organ Distribution of Gold Nanoparticles after Intravenous Administration. Biomaterials 29 (12), 1912–1919. 10.1016/j.biomaterials.2007.12.037 18242692

[B26] de Souza M. L. Dos Santos W. M. de Sousa A. L. M. D. de Albuquerque Wanderley Sales V. Nóbrega F. P. de Oliveira M. V. G. (2020). Lipid Nanoparticles as a Skin Wound Healing Drug Delivery System: Discoveries and Advances. Cpd 26 (36), 4536–4550. 10.2174/1381612826666200417144530 32303163

[B27] Debele T. A. Mekuria S. L. Tsai H.-C. (2016). Polysaccharide Based Nanogels in the Drug Delivery System: Application as the Carrier of Pharmaceutical Agents. Mater. Sci. Eng. C 68, 964–981. 10.1016/j.msec.2016.05.121 27524098

[B28] DeTure M. A. Dickson D. W. (2019). The Neuropathological Diagnosis of Alzheimer's Disease. Mol. Neurodegener. 14 (1), 32. 10.1186/s13024-019-0333-5 31375134 PMC6679484

[B29] Dowling J. Isbister G. K. Kirkpatrick C. M. J. Naidoo D. Graudins A. (2008). Population Pharmacokinetics of Intravenous, Intramuscular, and Intranasal Naloxone in Human Volunteers. Ther. Drug Monit. 30 (4), 490–496. 10.1097/ftd.0b013e3181816214 18641540

[B30] Dutta T. Jain N. K. (2007). Targeting Potential and Anti-HIV Activity of Lamivudine Loaded Mannosylated Poly (Propyleneimine) Dendrimer. Biochimica Biophysica Acta (BBA) - General Subj. 1770 (4), 681–686. 10.1016/j.bbagen.2006.12.007 17276009

[B31] Engelhardt B. Sorokin L. (2009). The Blood-Brain and the Blood-Cerebrospinal Fluid Barriers: Function and Dysfunction. Semin. Immunopathol. 31 (4), 497–511. 10.1007/s00281-009-0177-0 19779720

[B32] Erickson M. A. Banks W. A. (2018). Neuroimmune Axes of the Blood-Brain Barriers and Blood-Brain Interfaces: Bases for Physiological Regulation, Disease States, and Pharmacological Interventions. Pharmacol. Rev. 70 (2), 278–314. 10.1124/pr.117.014647 29496890 PMC5833009

[B33] Falagas M. E. Bliziotis I. A. Tam V. H. (2007). Intraventricular or Intrathecal Use of Polymyxins in Patients with Gram-Negative Meningitis: a Systematic Review of the Available Evidence. Int. J. Antimicrob. Agents 29 (1), 9–25. 10.1016/j.ijantimicag.2006.08.024 17126534

[B34] Fassas A. Anagnostopoulos A. (2005). The Use of Liposomal Daunorubicin (DaunoXome) in Acute Myeloid Leukemia. Leukemia Lymphoma 46 (6), 795–802. 10.1080/10428190500052438 16019523

[B35] Feng X. Xu W. Li Z. Song W. Ding J. Chen X. (2019). Immunomodulatory Nanosystems. Adv. Sci. 6 (17), 1900101. 10.1002/advs.201900101 PMC6724480 31508270

[B36] Francischi J. N. Pereira L. S. M. Castro M. S. (1997). Cyclosporin Inhibits Hyperalgesia and Edema in Arthritic Rats: Role of the Central Nervous System. Braz J. Med. Biol. Res. 30 (1), 101–111. 10.1590/s0100-879x1997000100016 9222411

[B37] Furtado D. Björnmalm M. Ayton S. Bush A. I. Kempe K. Caruso F. (2018). Overcoming the Blood-Brain Barrier: The Role of Nanomaterials in Treating Neurological Diseases. Adv. Mater 30 (46), e1801362. 10.1002/adma.201801362 30066406

[B38] Gadhave D. Rasal N. Sonawane R. Sekar M. Kokare C. (2021). Nose-to-brain Delivery of Teriflunomide-Loaded Lipid-Based Carbopol-Gellan Gum Nanogel for Glioma: Pharmacological and *In Vitro* Cytotoxicity Studies. Int. J. Biol. Macromol. 167, 906–920. 10.1016/j.ijbiomac.2020.11.047 33186648

[B39] Ganguly K. Chaturvedi K. More U. A. Nadagouda M. N. Aminabhavi T. M. (2014). Polysaccharide-based Micro/nanohydrogels for Delivering Macromolecular Therapeutics. J. Control. Release 193, 162–173. 10.1016/j.jconrel.2014.05.014 24845128

[B40] Gerson T. Makarov E. Senanayake T. H. Gorantla S. Poluektova L. Y. Vinogradov S. V. (2014). Nano-NRTIs Demonstrate Low Neurotoxicity and High Antiviral Activity against HIV Infection in the Brain. Nanomedicine Nanotechnol. Biol. Med. 10 (1), 177–185. 10.1016/j.nano.2013.06.012 PMC3843977 23845925

[B41] Ghersi-Egea J.-F. Strazielle N. Catala M. Silva-Vargas V. Doetsch F. Engelhardt B. (2018). Molecular Anatomy and Functions of the Choroidal Blood-Cerebrospinal Fluid Barrier in Health and Disease. Acta Neuropathol. 135 (3), 337–361. 10.1007/s00401-018-1807-1 29368213

[B42] Giacobini E. Gold G. (2013). Alzheimer Disease Therapy-Moving from Amyloid-β to Tau. Nat. Rev. Neurol. 9 (12), 677–686. 10.1038/nrneurol.2013.223 24217510

[B43] Gil-Martins E. Barbosa D. J. Silva V. Remião F. Silva R. (2020). Dysfunction of ABC Transporters at the Blood-Brain Barrier: Role in Neurological Disorders. Pharmacol. Ther. 213, 107554. 10.1016/j.pharmthera.2020.107554 32320731

[B44] Girolamo F. de Trizio I. Errede M. Longo G. d’Amati A. Virgintino D. (2021). Neural Crest Cell-Derived Pericytes Act as Pro-angiogenic Cells in Human Neocortex Development and Gliomas. Fluids Barriers CNS 18 (1), 14. 10.1186/s12987-021-00242-7 33743764 PMC7980348

[B45] Godinho B. M. D. C. Ogier J. R. Darcy R. O’Driscoll C. M. Cryan J. F. (2013). Self-assembling Modified β-Cyclodextrin Nanoparticles as Neuronal siRNA Delivery Vectors: Focus on Huntington's Disease. Mol. Pharm. 10 (2), 640–649. 10.1021/mp3003946 23116281

[B46] Goldberg M. Langer R. Jia X. (2007). Nanostructured Materials for Applications in Drug Delivery and Tissue Engineering. J. Biomaterials Sci. Polym. Ed. 18 (3), 241–268. 10.1163/156856207779996931 PMC3017754 17471764

[B47] Gong B. Zhang X. Zahrani A. A. Gao W. Ma G. Zhang L. (2022). Neural Tissue Engineering: From Bioactive Scaffolds and *In Situ* Monitoring to Regeneration. Exploration 1, 1. 10.1002/EXP.20210035 PMC10190951 37323703

[B48] Grassin-Delyle S. Buenestado A. Naline E. Faisy C. Blouquit-Laye S. Couderc L.-J. (2012). Intranasal Drug Delivery: An Efficient and Non-invasive Route for Systemic Administration. Pharmacol. Ther. 134 (3), 366–379. 10.1016/j.pharmthera.2012.03.003 22465159

[B49] Guo D. Lou C. Wang N. Chen M. Zhang P. Wu S. (2017). Poly (Styrene-divinyl Benzene-Glycidylmethacrylate) Stationary Phase Grafted with Poly Amidoamine (PAMAM) Dendrimers for Rapid Determination of Phenylene Diamine Isomers in HPLC. Talanta 168, 188–195. 10.1016/j.talanta.2017.03.053 28391841

[B51] Hanada T. (2014). The Discovery and Development of Perampanel for the Treatment of Epilepsy. Expert Opin. Drug Discov. 9 (4), 449–458. 10.1517/17460441.2014.891580 24559052

[B52] Harbi I. Aljaeid B. El-Say K. M. Zidan A. S. (2016). Glycosylated Sertraline-Loaded Liposomes for Brain Targeting: QbD Study of Formulation Variabilities and Brain Transport. AAPS PharmSciTech 17 (6), 1404–1420. 10.1208/s12249-016-0481-7 26786680

[B53] Hawkins B. T. Davis T. P. (2005). The Blood-Brain Barrier/neurovascular Unit in Health and Disease. Pharmacol. Rev. 57 (2), 173–185. 10.1124/pr.57.2.4 15914466

[B54] Hawthorne G. H. Bernuci M. P. Bortolanza M. Tumas V. Issy A. C. Del-Bel E. (2016). Nanomedicine to Overcome Current Parkinson's Treatment Liabilities: A Systematic Review. Neurotox. Res. 30 (4), 715–729. 10.1007/s12640-016-9663-z 27581037

[B55] He Q. Liu J. Liang J. Liu X. Li W. Liu Z. (2018). Towards Improvements for Penetrating the Blood-Brain Barrier-Recent Progress from a Material and Pharmaceutical Perspective. Cells 7 (4), 24. 10.3390/cells7040024 PMC5946101 29570659

[B56] He W. Zhang Z. Sha X. (2021). Nanoparticles-mediated Emerging Approaches for Effective Treatment of Ischemic Stroke. Biomaterials 277, 121111. 10.1016/j.biomaterials.2021.121111 34488117

[B57] Heldt T. Zoerle T. Teichmann D. Stocchetti N. (2019). Intracranial Pressure and Intracranial Elastance Monitoring in Neurocritical Care. Annu. Rev. Biomed. Eng. 21, 523–549. 10.1146/annurev-bioeng-060418-052257 31167100

[B58] Houng A. K. Wang D. Reed G. L. (2014). Reversing the Deleterious Effects of α2-antiplasmin on Tissue Plasminogen Activator Therapy Improves Outcomes in Experimental Ischemic Stroke. Exp. Neurol. 255, 56–62. 10.1016/j.expneurol.2014.02.009 24556477 PMC4066326

[B59] Huang K. Shi B. Xu W. Ding J. Yang Y. Liu H. (2015). Reduction-responsive Polypeptide Nanogel Delivers Antitumor Drug for Improved Efficacy and Safety. Acta Biomater. 27, 179–193. 10.1016/j.actbio.2015.08.049 26320542

[B60] Huo T. Barth R. F. Yang W. Nakkula R. J. Koynova R. Tenchov B. (2012). Preparation, Biodistribution and Neurotoxicity of Liposomal Cisplatin Following Convection Enhanced Delivery in Normal and F98 Glioma Bearing Rats. PLoS One 7 (11), e48752. 10.1371/journal.pone.0048752 23152799 PMC3496719

[B61] Jafari S. Derakhshankhah H. Alaei L. Fattahi A. Varnamkhasti B. S. Saboury A. A. (2019). Mesoporous Silica Nanoparticles for Therapeutic/diagnostic Applications. Biomed. Pharmacother. 109, 1100–1111. 10.1016/j.biopha.2018.10.167 30551360

[B62] Jafarieh O. Md S. Ali M. Baboota S. Sahni J. K. Kumari B. (2015). Design, Characterization, and Evaluation of Intranasal Delivery of Ropinirole-Loaded Mucoadhesive Nanoparticles for Brain Targeting. Drug Dev. Industrial Pharm. 41 (10), 1674–1681. 10.3109/03639045.2014.991400 25496439

[B63] Jain K. K. (2020). An Overview of Drug Delivery Systems. Methods Mol. Biol. 2059, 1–54. 10.1007/978-1-4939-9798-5_1 31435914

[B64] Jain S. Malinowski M. Chopra P. Varshney V. Deer T. R. (2019). Intrathecal Drug Delivery for Pain Management: Recent Advances and Future Developments. Expert Opin. Drug Deliv. 16 (8), 815–822. 10.1080/17425247.2019.1642870 31305165

[B65] Javed B. Zhao X. Cui D. Curtin J. Tian F. (2021). Enhanced Anticancer Response of Curcumin- and Piperine-Loaded Lignin-G-P (NIPAM-Co-DMAEMA) Gold Nanogels against U-251 MG Glioblastoma Multiforme. Biomedicines 9 (11), 1516. 10.3390/biomedicines9111516 34829745 PMC8615061

[B66] Jiang Z. Dong X. Yan X. Liu Y. Zhang L. Sun Y. (2018). Nanogels of Dual Inhibitor-Modified Hyaluronic Acid Function as a Potent Inhibitor of Amyloid β-protein Aggregation and Cytotoxicity. Sci. Rep. 8 (1), 3505. 10.1038/s41598-018-21933-6 29472606 PMC5823891

[B67] Jin J. Bae K. H. Yang H. Lee S. J. Kim H. Kim Y. (2011). *In Vivo* specific Delivery of C-Met siRNA to Glioblastoma Using Cationic Solid Lipid Nanoparticles. Bioconjugate Chem. 22 (12), 2568–2572. 10.1021/bc200406n 22070554

[B68] Jogani V. Jinturkar K. Vyas T. Misra A. (2008). Recent Patents Review on Intranasal Administration for CNS Drug Delivery. Recent Pat. Drug Deliv. Formul. 2 (1), 25–40. 10.2174/187221108783331429 19075895

[B69] Johanson C. Stopa E. McMillan P. Roth D. Funk J. Krinke G. (2011). The Distributional Nexus of Choroid Plexus to Cerebrospinal Fluid, Ependyma and Brain. Toxicol. Pathol. 39 (1), 186–212. 10.1177/0192623310394214 21189316

[B70] Kadry H. Noorani B. Cucullo L. (2020). A Blood-Brain Barrier Overview on Structure, Function, Impairment, and Biomarkers of Integrity. Fluids Barriers CNS 17 (1), 69. 10.1186/s12987-020-00230-3 33208141 PMC7672931

[B71] Kamaly N. Xiao Z. Valencia P. M. Radovic-Moreno A. F. Farokhzad O. C. (2012). Targeted Polymeric Therapeutic Nanoparticles: Design, Development and Clinical Translation. Chem. Soc. Rev. 41 (7), 2971–3010. 10.1039/c2cs15344k 22388185 PMC3684255

[B72] Kazkayasi I. Telli G. Nemutlu E. Uma S. (2022). Intranasal Metformin Treatment Ameliorates Cognitive Functions via Insulin Signaling Pathway in ICV-STZ-Induced Mice Model of Alzheimer's Disease. Life Sci. 299, 120538. 10.1016/j.lfs.2022.120538 35395244

[B73] Khan A. R. Yang X. Fu M. Zhai G. (2018). Recent Progress of Drug Nanoformulations Targeting to Brain. J. Control. Release 291, 37–64. 10.1016/j.jconrel.2018.10.004 30308256

[B74] Khan O. Chaudary N. (2020). The Use of Amikacin Liposome Inhalation Suspension (Arikayce) in the Treatment of Refractory Nontuberculous Mycobacterial Lung Disease in Adults. Dddt 14, 2287–2294. 10.2147/dddt.s146111 32606598 PMC7293904

[B75] Kim M. Kizilbash S. H. Laramy J. K. Gampa G. Parrish K. E. Sarkaria J. N. (2018). Barriers to Effective Drug Treatment for Brain Metastases: A Multifactorial Problem in the Delivery of Precision Medicine. Pharm. Res. 35 (9), 177. 10.1007/s11095-018-2455-9 30003344 PMC6700736

[B76] Kleindorfer D. O. Khatri P. Katzan I. (2005). Reasons for Exclusion from Thrombolytic Therapy Following Acute Ischemic Stroke. Neurology 65 (11), 1844. 10.1212/01.wnl.0000200031.41939.ac 16353320

[B77] Kreyling W. G. Hirn S. Möller W. Schleh C. Wenk A. Celik G. (2014). Air-blood Barrier Translocation of Tracheally Instilled Gold Nanoparticles Inversely Depends on Particle Size. ACS Nano 8 (1), 222–233. 10.1021/nn403256v 24364563 PMC3960853

[B78] Kumar A. Tan A. Wong J. Spagnoli J. C. Lam J. Blevins B. D. (2017). Nanotechnology for Neuroscience: Promising Approaches for Diagnostics, Therapeutics and Brain Activity Mapping. Adv. Funct. Mater 27 (39), 1700489. 10.1002/adfm.201700489 30853878 PMC6404766

[B79] Kuo Y.-C. Shih-Huang C.-Y. (2013). Solid Lipid Nanoparticles Carrying Chemotherapeutic Drug across the Blood-Brain Barrier through Insulin Receptor-Mediated Pathway. J. Drug Target. 21 (8), 730–738. 10.3109/1061186x.2013.812094 23815407

[B80] Lee G. Dallas S. Hong M. Bendayan R. (2001). Drug Transporters in the Central Nervous System: Brain Barriers and Brain Parenchyma Considerations. Pharmacol. Rev. 53 (4), 569–596. 11734619

[B81] Lee K. Kim T. Kim Y. M. Yang K. Choi I. Roh Y. H. (2021). Multifunctional DNA Nanogels for Aptamer-Based Targeted Delivery and Stimuli-Triggered Release of Cancer Therapeutics. Macromol. Rapid Commun. 42 (2), e2000457. 10.1002/marc.202000457 33230833

[B82] Leoni V. Caccia C. (2014). Study of Cholesterol Metabolism in Huntington′s Disease. Biochem. Biophysical Res. Commun. 446 (3), 697–701. 10.1016/j.bbrc.2014.01.188 24525128

[B83] Li D. Mastaglia F. L. Fletcher S. Wilton S. D. (2020). Progress in the Molecular Pathogenesis and Nucleic Acid Therapeutics for Parkinson's Disease in the Precision Medicine Era. Med. Res. Rev. 40 (6), 2650–2681. 10.1002/med.21718 32767426 PMC7589267

[B84] Liu C. Wen J. Li D. Qi H. Nih L. Zhu J. (2021). Systemic Delivery of microRNA for Treatment of Brain Ischemia. Nano Res. 14 (9), 3319–3328. 10.1007/s12274-021-3413-8

[B85] Liu J. Liu C. Zhang J. Zhang Y. Liu K. Song J.-X. (2020). A Self-Assembled α-Synuclein Nanoscavenger for Parkinson's Disease. ACS Nano 14 (2), 1533–1549. 10.1021/acsnano.9b06453 32027482

[B86] Liu Y. Li D. Ding J. Chen X. (2020). Controlled Synthesis of Polypeptides. Chin. Chem. Lett. 31 (12), 3001–3014. 10.1016/j.cclet.2020.04.029

[B87] Liu Z. Qiao J. Nagy T. Xiong M. P. (2018). ROS-Triggered Degradable Iron-Chelating Nanogels: Safely Improving Iron Elimination *In Vivo* . J. Control. Release 283, 84–93. 10.1016/j.jconrel.2018.05.025 29792889 PMC6035766

[B88] Logan S. Arzua T. Canfield S. G. Seminary E. R. Sison S. L. Ebert A. D. (2019). Studying Human Neurological Disorders Using Induced Pluripotent Stem Cells: From 2D Monolayer to 3D Organoid and Blood Brain Barrier Models. Compr. Physiol. 9 (2), 565–611. 10.1002/cphy.c180025 30873582 PMC6705133

[B89] Lombardo D. Calandra P. Pasqua L. Magazù S. (2020). Self-assembly of Organic Nanomaterials and Biomaterials: The Bottom-Up Approach for Functional Nanostructures Formation and Advanced Applications. Mater. (Basel) 13 (5), 1048. 10.3390/ma13051048 PMC7084717 32110877

[B90] Löscher W. Potschka H. Sisodiya S. M. Vezzani A. (2020). Drug Resistance in Epilepsy: Clinical Impact, Potential Mechanisms, and New Innovative Treatment Options. Pharmacol. Rev. 72 (3), 606–638. 10.1124/pr.120.019539 32540959 PMC7300324

[B91] Ma Y. Ge Y. Li L. (2017). Advancement of Multifunctional Hybrid Nanogel Systems: Construction and Application in Drug Co-delivery and Imaging Technique. Mater. Sci. Eng. C 71, 1281–1292. 10.1016/j.msec.2016.11.031 27987684

[B92] Mahajan H. S. Mahajan M. S. Nerkar P. P. Agrawal A. (2014). Nanoemulsion-based Intranasal Drug Delivery System of Saquinavir Mesylate for Brain Targeting. Drug Deliv. 21 (2), 148–154. 10.3109/10717544.2013.838014 24128122

[B93] Mao L. Wang H. Tan M. Ou L. Kong D. Yang Z. (2012). Conjugation of Two Complementary Anti-cancer Drugs Confers Molecular Hydrogels as a Co-delivery System. Chem. Commun. 48 (3), 395–397. 10.1039/c1cc16250k 22080052

[B94] Marçal H. Ahmed T. Badylak S. F. Tottey S. Foster L. J. R. (2012). A Comprehensive Protein Expression Profile of Extracellular Matrix Biomaterial Derived from Porcine Urinary Bladder. Regen. Med. 7 (2), 159–166. 10.2217/rme.12.6 22397606

[B95] Marí M. Morales A. Colell A. García-Ruiz C. Fernández-Checa J. C. (2009). Mitochondrial Glutathione, a Key Survival Antioxidant. Antioxidants Redox Signal. 11 (11), 2685–2700. 10.1089/ars.2009.2695 PMC2821140 19558212

[B96] Mathew A. P. Uthaman S. Cho K.-H. Cho C.-S. Park I.-K. (2018). Injectable Hydrogels for Delivering Biotherapeutic Molecules. Int. J. Biol. Macromol. 110, 17–29. 10.1016/j.ijbiomac.2017.11.113 29169942

[B97] Mauri E. Giannitelli S. M. Trombetta M. Rainer A. (2021). Synthesis of Nanogels: Current Trends and Future Outlook. Gels 7 (2), 36. 10.3390/gels7020036 33805279 PMC8103252

[B98] McAteer J. A. Evan A. P. (2008). The Acute and Long-Term Adverse Effects of Shock Wave Lithotripsy. Seminars Nephrol. 28 (2), 200–213. 10.1016/j.semnephrol.2008.01.003 PMC2900184 18359401

[B99] McKee A. C. Robinson M. E. (2014). Military-related Traumatic Brain Injury and Neurodegeneration. Alzheimers Dement. 10 (3 Suppl. l), S242–S253. 10.1016/j.jalz.2014.04.003 24924675 PMC4255273

[B100] McNaught K. S. P. Belizaire R. Jenner P. Olanow C. W. Isacson O. (2002). Selective Loss of 20S Proteasome α-subunits in the Substantia Nigra Pars Compacta in Parkinson's Disease. Neurosci. Lett. 326 (3), 155–158. 10.1016/s0304-3940(02)00296-3 12095645

[B101] Meairs S. (2015). Facilitation of Drug Transport across the Blood-Brain Barrier with Ultrasound and Microbubbles. Pharmaceutics 7 (3), 275–293. 10.3390/pharmaceutics7030275 26404357 PMC4588200

[B102] Medina S. H. El-Sayed M. E. H. (2009). Dendrimers as Carriers for Delivery of Chemotherapeutic Agents. Chem. Rev. 109 (7), 3141–3157. 10.1021/cr900174j 19534493

[B103] Meng X.-y. Li J.-j. Ni T.-j. Xiao-tong L. He T. Men Z.-n. (2020). Electro-responsive Brain-Targeting Mixed Micelles Based on Pluronic F127 and D-α-Tocopherol Polyethylene Glycol Succinate–Ferrocene. Colloids Surfaces A Physicochem. Eng. Aspects 601, 124986. 10.1016/j.colsurfa.2020.124986

[B104] Merino S. Martín C. Kostarelos K. Prato M. Vázquez E. (2015). Nanocomposite Hydrogels: 3D Polymer-Nanoparticle Synergies for On-Demand Drug Delivery. ACS Nano 9 (5), 4686–4697. 10.1021/acsnano.5b01433 25938172

[B105] Mihalko E. P. Nellenbach K. Krishnakumar M. Moiseiwitsch N. Sollinger J. Cooley B. C. (2022). Fibrin-specific poly(N-Isopropylacrylamide) Nanogels for Targeted Delivery of Tissue-type Plasminogen Activator to Treat Thrombotic Complications Are Well Tolerated *In Vivo* . Bioeng. Transl. Med. 7 (2), e10277. 10.1002/btm2.10277 35600656 PMC9115681

[B106] Misra A. Ganesh S. Shahiwala A. Shah S. P. (2003). Drug Delivery to the Central Nervous System: a Review. J. Pharm. Pharm. Sci. 6 (2), 252–273. 12935438

[B107] Molino Y. Jabès F. Lacassagne E. Gaudin N. Khrestchatisky M. (2014). Setting-up an *In Vitro* Model of Rat Blood-Brain Barrier (BBB): a Focus on BBB Impermeability and Receptor-Mediated Transport. J. Vis. Exp. 88, e51278. 10.3791/51278 PMC4208856 24998179

[B108] Muresanu D. F. Strilciuc S. Stan A. (2019). Current Drug Treatment of Acute Ischemic Stroke: Challenges and Opportunities. CNS Drugs 33 (9), 841–847. 10.1007/s40263-019-00663-x 31512153

[B109] Nance E. Zhang C. Shih T.-Y. Xu Q. Schuster B. S. Hanes J. (2014). Brain-penetrating Nanoparticles Improve Paclitaxel Efficacy in Malignant Glioma Following Local Administration. ACS Nano 8 (10), 10655–10664. 10.1021/nn504210g 25259648 PMC4212792

[B110] Nau R. Sörgel F. Eiffert H. (2021). Central Nervous System Infections and Antimicrobial Resistance: an Evolving Challenge. Curr. Opin. Neurol. 34 (3), 456–467. 10.1097/wco.0000000000000931 33767092

[B111] Neamtu I. Rusu A. G. Diaconu A. Nita L. E. Chiriac A. P. (2017). Basic Concepts and Recent Advances in Nanogels as Carriers for Medical Applications. Drug Deliv. 24 (1), 539–557. 10.1080/10717544.2016.1276232 28181831 PMC8240973

[B112] Oberdörster G. (2010). Safety Assessment for Nanotechnology and Nanomedicine: Concepts of Nanotoxicology. J. Intern Med. 267 (1), 89–105. 10.1111/j.1365-2796.2009.02187.x 20059646

[B113] Oh J. K. Siegwart D. J. Matyjaszewski K. (2007). Synthesis and Biodegradation of Nanogels as Delivery Carriers for Carbohydrate Drugs. Biomacromolecules 8 (11), 3326–3331. 10.1021/bm070381+ 17894465

[B114] Palmer A. M. (2010). The Role of the Blood-CNS Barrier in CNS Disorders and Their Treatment. Neurobiol. Dis. 37 (1), 3–12. 10.1016/j.nbd.2009.07.029 19664711

[B115] Pappu S. Lerma J. Khraishi T. (2016). Brain CT to Assess Intracranial Pressure in Patients with Traumatic Brain Injury. J. Neuroimaging 26 (1), 37–40. 10.1111/jon.12289 26752449

[B116] Pardridge W. M. (2003). Blood-brain Barrier Drug Targeting: the Future of Brain Drug Development. Mol. Interv. 3 (2), 9051–9105. 10.1124/mi.3.2.90 14993430

[B117] Pardridge W. M. (2005). The Blood-Brain Barrier: Bottleneck in Brain Drug Development. Neurotherapeutics 2 (1), 3–14. 10.1602/neurorx.2.1.3 PMC539316 15717053

[B118] Pargoo E. M. Aghasadeghi M. R. Parivar K. Nikbin M. Rahimi P. Ardestani M. S. (2021). Lamivudine‐conjugated and Efavirenz‐loaded G2 Dendrimers: Novel Anti‐retroviral Nano Drug Delivery Systems. IET Nanobiotechnol. 15 (7), 627–637. 10.1049/nbt2.12060 34695297 PMC8675833

[B119] Pastor-Maldonado C. J. Suárez-Rivero J. M. Povea-Cabello S. Álvarez-Córdoba M. Villalón-García I. Munuera-Cabeza M. (2020). Coenzyme Q10: Novel Formulations and Medical Trends. Int. J. Mol. Sci. 21 (22), 8432. 10.3390/ijms21228432 PMC7697799 33182646

[B120] Patel V. Chavda V. Shah J. (2021). Nanotherapeutics in Neuropathologies: Obstacles, Challenges and Recent Advancements in CNS Targeted Drug Delivery Systems. Curr. Neuropharmacol. 19 (5), 693–710. 10.2174/1570159x18666200807143526 32851949 PMC8573747

[B121] Peng S. Wang H. Zhao W. Xin Y. Liu Y. Yu X. (2020). Zwitterionic Polysulfamide Drug Nanogels with Microwave Augmented Tumor Accumulation and On‐Demand Drug Release for Enhanced Cancer Therapy. Adv. Funct. Mater. 30 (23), 1. 10.1002/adfm.202001832

[B122] Pichla M. Bartosz G. Sadowska-Bartosz I. (2020). The Antiaggregative and Antiamyloidogenic Properties of Nanoparticles: A Promising Tool for the Treatment and Diagnostics of Neurodegenerative Diseases. Oxid. Med. Cell Longev. 2020, 3534570. 10.1155/2020/3534570 33123310 PMC7582079

[B123] Picone P. Sabatino M. A. Ditta L. A. Amato A. San Biagio P. L. Mulè F. (2018). Nose-to-brain Delivery of Insulin Enhanced by a Nanogel Carrier. J. Control. Release 270, 23–36. 10.1016/j.jconrel.2017.11.040 29196041

[B124] Poovaiah N. Davoudi Z. Peng H. Schlichtmann B. Mallapragada S. Narasimhan B. (2018). Treatment of Neurodegenerative Disorders through the Blood-Brain Barrier Using Nanocarriers. Nanoscale 10 (36), 16962–16983. 10.1039/c8nr04073g 30182106

[B125] Pottoo F. H. Sharma S. Javed M. N. Barkat M. A. Harshita Alam M. S. (2020). Lipid-based Nanoformulations in the Treatment of Neurological Disorders. Drug Metab. Rev. 52 (1), 185–204. 10.1080/03602532.2020.1726942 32116044

[B126] Pourtalebi Jahromi L. Moghaddam Panah F. Azadi A. Ashrafi H. (2019). A Mechanistic Investigation on Methotrexate-Loaded Chitosan-Based Hydrogel Nanoparticles Intended for CNS Drug Delivery: Trojan Horse Effect or Not? Int. J. Biol. Macromol. 125, 785–790. 10.1016/j.ijbiomac.2018.12.093 30543880

[B127] Pourtalebi Jahromi L. Mohammadi-Samani S. Heidari R. Azadi A. (2018). In Vitro- and *In Vivo* Evaluation of Methotrexate-Loaded Hydrogel Nanoparticles Intended to Treat Primary CNS Lymphoma via Intranasal Administration. J. Pharm. Pharm. Sci. 21 (1), 305–317. 10.18433/jpps29496 30053381

[B128] Prasanna P. Upadhyay A. (2021). Flavonoid-Based Nanomedicines in Alzheimer's Disease Therapeutics: Promises Made, a Long Way to Go. ACS Pharmacol. Transl. Sci. 4 (1), 74–95. 10.1021/acsptsci.0c00224 33615162 PMC7887745

[B129] Rizzi L. Rosset I. Roriz-Cruz M. (2014). Global Epidemiology of Dementia: Alzheimer's and Vascular Types. Biomed. Res. Int. 2014, 908915. 10.1155/2014/908915 25089278 PMC4095986

[B130] Sá-Pereira I. Brites D. Brito M. A. (2012). Neurovascular Unit: a Focus on Pericytes. Mol. Neurobiol. 45 (2), 327–347. 10.1007/s12035-012-8244-2 22371274

[B131] Saha P. Ganguly R. Li X. Das R. Singha N. K. Pich A. (2021). Zwitterionic Nanogels and Microgels: An Overview on Their Synthesis and Applications. Macromol. Rapid Commun. 42 (13), e2100112. 10.1002/marc.202100112 34021658

[B132] Sari N. D. Baltali S. Serin I. Antar V. (2021). Evaluation of Intraventricular/Intrathecal Antimicrobial Therapy in the Treatment of Nosocomial Meningitis Caused by Multidrug-Resistant Gram-Negative Bacteria after Central Nervous System Surgery. Can. J. Infect. Dis. Med. Microbiol. 2021, 9923015. 10.1155/2021/9923015 34497678 PMC8419485

[B133] Sarkar A. Fatima I. Mohammad Sajid Jamal Q. Sayeed U. Kalim A. Khan M. Akhtar S. (2017). Nanoparticles as a Carrier System for Drug Delivery across Blood Brain Barrier. Cdm 18 (2), 129–137. 10.2174/1389200218666170113125132 28088890

[B134] Shi B. Huang K. Ding J. Xu W. Yang Y. Liu H. (2017). Intracellularly Swollen Polypeptide Nanogel Assists Hepatoma Chemotherapy. Theranostics 7 (3), 703–716. 10.7150/thno.16794 28255361 PMC5327644

[B135] Shringarpure M. Gharat S. Momin M. Omri A. (2021). Management of Epileptic Disorders Using Nanotechnology-Based Strategies for Nose-To-Brain Drug Delivery. Expert Opin. Drug Deliv. 18 (2), 169–185. 10.1080/17425247.2021.1823965 32921169

[B136] Soni K. S. Desale S. S. Bronich T. K. (2016). Nanogels: An Overview of Properties, Biomedical Applications and Obstacles to Clinical Translation. J. Control. Release 240, 109–126. 10.1016/j.jconrel.2015.11.009 26571000 PMC4862943

[B137] Soni S. Ruhela R. K. Medhi B. (2016). Nanomedicine in Central Nervous System (CNS) Disorders: A Present and Future Prospective. Adv. Pharm. Bull. 6 (3), 319–335. 10.15171/apb.2016.044 27766216 PMC5071795

[B138] Soni V. Jain A. Khare P. Gulbake A. Jain S. (2010). Potential Approaches for Drug Delivery to the Brain: Past, Present, and Future. Crit. Rev. Ther. Drug Carr. Syst. 27 (3), 187–236. 10.1615/critrevtherdrugcarriersyst.v27.i3.10 20540687

[B139] Srikanth M. Kessler J. A. (2012). Nanotechnology-novel Therapeutics for CNS Disorders. Nat. Rev. Neurol. 8 (6), 307–318. 10.1038/nrneurol.2012.76 22526003 PMC3773927

[B140] Stawicki B. Schacher T. Cho H. (2021). Nanogels as a Versatile Drug Delivery System for Brain Cancer. Gels 7 (2), 63. 10.3390/gels7020063 34073626 PMC8162335

[B141] Stuart M. A. C. Huck W. T. S. Genzer J. Müller M. Ober C. Stamm M. (2010). Emerging Applications of Stimuli-Responsive Polymer Materials. Nat. Mater 9 (2), 101–113. 10.1038/nmat2614 20094081

[B142] Suhail M. Rosenholm J. M. Minhas M. U. Badshah S. F. Naeem A. Khan K. U. (2019). Nanogels as Drug-Delivery Systems: a Comprehensive Overview. Ther. Deliv. 10 (11), 697–717. 10.4155/tde-2019-0010 31789106

[B143] Sun M. Su X. Ding B. He X. Liu X. Yu A. (2012). Advances in Nanotechnology-Based Delivery Systems for Curcumin. Nanomedicine 7 (7), 1085–1100. 10.2217/nnm.12.80 22846093

[B144] Sun T. Zhang Y. S. Pang B. Hyun D. C. Yang M. Xia Y. (2014). Engineered Nanoparticles for Drug Delivery in Cancer Therapy. Angew. Chem. Int. Ed. Engl. 53 (46), 12320–12364. 10.1002/anie.201403036 25294565

[B146] Tang L. Zhang M. Liu C. (2022). Advances in Nanotechnology-Based Immunotherapy for Glioblastoma. Front. Immunol. 13, 882257. 10.3389/fimmu.2022.882257 35651605 PMC9149074

[B147] Tangpong J. Cole M. P. Sultana R. Estus S. Vore M. St. Clair W. (2007). Adriamycin-mediated Nitration of Manganese Superoxide Dismutase in the Central Nervous System: Insight into the Mechanism of Chemobrain. J. Neurochem. 100 (1), 191–201. 10.1111/j.1471-4159.2006.04179.x 17227439

[B148] Tedford C. E. DeLapp S. Jacques S. Anders J. (2015). Quantitative Analysis of Transcranial and Intraparenchymal Light Penetration in Human Cadaver Brain Tissue. Lasers Surg. Med. 47 (4), 312–322. 10.1002/lsm.22343 25772014

[B149] Teng Y. Jin H. Nan D. Li M. Fan C. Liu Y. (2018). *In Vivo* evaluation of Urokinase-Loaded Hollow Nanogels for Sonothrombolysis on Suture Embolization-Induced Acute Ischemic Stroke Rat Model. Bioact. Mater. 3 (1), 102–109. 10.1016/j.bioactmat.2017.08.001 29744447 PMC5935765

[B150] Tian X. H. Lin X. N. Wei F. Feng W. Huang Z. C. Wang P. (2011). Enhanced Brain Targeting of Temozolomide in Polysorbate-80 Coated Polybutylcyanoacrylate Nanoparticles. Int. J. Nanomedicine 6, 445–452. 10.2147/IJN.S16570 21445277 PMC3061435

[B151] Tietz S. Engelhardt B. (2015). Brain Barriers: Crosstalk between Complex Tight Junctions and Adherens Junctions. J. Cell Biol. 209 (4), 493–506. 10.1083/jcb.201412147 26008742 PMC4442813

[B152] Trompetero A. Gordillo A. Del Pilar M. C. Cristina V. M. Bustos Cruz R. H. (2018). Alzheimer's Disease and Parkinson's Disease: A Review of Current Treatment Adopting a Nanotechnology Approach. Cpd 24 (1), 22–45. 10.2174/1381612823666170828133059 28847307

[B153] T. Ronaldson P. P. Davis T. (2012). Blood-brain Barrier Integrity and Glial Support: Mechanisms that Can Be Targeted for Novel Therapeutic Approaches in Stroke. Curr. Pharm. Des. 18 (25), 3624–3644. 10.2174/138161212802002625 22574987 PMC3918413

[B154] Ueno M. Nakagawa T. Wu B. Onodera M. Huang C.-l. Kusaka T. (2010). Transporters in the Brain Endothelial Barrier. Cmc 17 (12), 1125–1138. 10.2174/092986710790827816 20175745

[B155] Verma S. Domb A. J. Kumar N. (2011). Nanomaterials for Regenerative Medicine. Nanomedicine 6 (1), 157–181. 10.2217/nnm.10.146 21182426

[B156] Vigani B. Rossi S. Sandri G. Bonferoni M. C. Caramella C. M. Ferrari F. (2020). Recent Advances in the Development of *In Situ* Gelling Drug Delivery Systems for Non-parenteral Administration Routes. Pharmaceutics 12 (9), 859. 10.3390/pharmaceutics12090859 PMC7559482 32927595

[B157] Vinogradov S. V. Batrakova E. V. Kabanov A. V. (2004). Nanogels for Oligonucleotide Delivery to the Brain. Bioconjugate Chem. 15 (1), 50–60. 10.1021/bc034164r PMC2837941 14733583

[B158] Vinogradov S. V. Bronich T. K. Kabanov A. V. (2002). Nanosized Cationic Hydrogels for Drug Delivery: Preparation, Properties and Interactions with Cells. Adv. Drug Deliv. Rev. 54 (1), 135–147. 10.1016/s0169-409x(01)00245-9 11755709

[B159] Vinogradov S. V. (2010). Nanogels in the Race for Drug Delivery. Nanomedicine 5 (2), 165–168. 10.2217/nnm.09.103 20148627

[B160] Vinogradov S. V. Poluektova L. Y. Makarov E. Gerson T. Senanayake M. T. (2010). Nano-NRTIs: Efficient Inhibitors of HIV Type-1 in Macrophages with a Reduced Mitochondrial Toxicity. Antivir. Chem. Chemother. 21 (1), 1–14. 10.3851/imp1680 21045256 PMC3032393

[B161] Wang D. Wu L.-P. (2017). Nanomaterials for Delivery of Nucleic Acid to the Central Nervous System (CNS). Mater. Sci. Eng. C 70 (Pt 2), 1039–1046. 10.1016/j.msec.2016.04.011 27772703

[B162] Wang J. Ni Q. Wang Y. Zhang Y. He H. Gao D. (2021). Nanoscale Drug Delivery Systems for Controllable Drug Behaviors by Multi-Stage Barrier Penetration. J. Control. Release 331, 282–295. 10.1016/j.jconrel.2020.08.045 32866590

[B163] Wang L. Lian W. Liu B. Lv H. Zhang Y. Wu X. (2022). Transparent, High-Performance and Stable Sb2 S3 Photoanode Enabled by Heterojunction Engineering with Conjugated Polycarbazole Frameworks for Unbiased Photoelectrochemical Overall Water Splitting Devices. Adv. Mater 1, e2200723. 10.1002/adma.202200723 35580906

[B164] Wang X. Parvathaneni V. Shukla S. K. Kulkarni N. S. Muth A. Kunda N. K. (2020). Inhalable Resveratrol-Cyclodextrin Complex Loaded Biodegradable Nanoparticles for Enhanced Efficacy against Non-small Cell Lung Cancer. Int. J. Biol. Macromol. 164, 638–650. 10.1016/j.ijbiomac.2020.07.124 32693132

[B165] Wang Y.-Y. Lui P. C. Li J. Y. (2009). Receptor-mediated Therapeutic Transport across the Blood-Brain Barrier. Immunotherapy 1 (6), 983–993. 10.2217/imt.09.75 20635914

[B166] Wang Y. Ying X. Chen L. Liu Y. Wang Y. Liang J. (2016). Electroresponsive Nanoparticles Improve Antiseizure Effect of Phenytoin in Generalized Tonic-Clonic Seizures. Neurotherapeutics 13 (3), 603–613. 10.1007/s13311-016-0431-9 27137202 PMC4965401

[B167] Warren G. Makarov E. Lu Y. Senanayake T. Rivera K. Gorantla S. (2015). Amphiphilic Cationic Nanogels as Brain-Targeted Carriers for Activated Nucleoside Reverse Transcriptase Inhibitors. J. Neuroimmune Pharmacol. 10 (1), 88–101. 10.1007/s11481-014-9576-7 25559020 PMC4336642

[B168] Wei P. Cornel E. J. Du J. (2021). Ultrasound-responsive Polymer-Based Drug Delivery Systems. Drug Deliv. Transl. Res. 11 (4), 1323–1339. 10.1007/s13346-021-00963-0 33761101 PMC7989687

[B169] Wen J. Wu D. Qin M. Liu C. Wang L. Xu D. (2019). Sustained Delivery and Molecular Targeting of a Therapeutic Monoclonal Antibody to Metastases in the Central Nervous System of Mice. Nat. Biomed. Eng. 3 (9), 706–716. 10.1038/s41551-019-0434-z 31384008 PMC6736720

[B170] Wilson L. Stewart W. Dams-O'Connor K. Diaz-Arrastia R. Horton L. Menon D. K. (2017). The Chronic and Evolving Neurological Consequences of Traumatic Brain Injury. Lancet Neurology 16 (10), 813–825. 10.1016/s1474-4422(17)30279-x 28920887 PMC9336016

[B171] Wohlfart S. Gelperina S. Kreuter J. (2012). Transport of Drugs across the Blood-Brain Barrier by Nanoparticles. J. Control. Release 161 (2), 264–273. 10.1016/j.jconrel.2011.08.017 21872624

[B172] Wong H. L. Wu X. Y. Bendayan R. (2012). Nanotechnological Advances for the Delivery of CNS Therapeutics. Adv. Drug Deliv. Rev. 64 (7), 686–700. 10.1016/j.addr.2011.10.007 22100125

[B174] Wu T. Tang M. (2018). The Inflammatory Response to Silver and Titanium Dioxide Nanoparticles in the Central Nervous System. Nanomedicine 13 (2), 233–249. 10.2217/nnm-2017-0270 29199887

[B175] Xiao Y. Liu F. Yang J. Zhong M. Zhang E. Li Y. (2015). Over-activation of TLR5 Signaling by High-Dose Flagellin Induces Liver Injury in Mice. Cell Mol. Immunol. 12 (6), 729–742. 10.1038/cmi.2014.110 25418468 PMC4716618

[B176] Xu C. Gong Y. Wang Y. Chen Z. (2022). New Advances in Pharmacoresistant Epilepsy towards Precise Management-From Prognosis to Treatments. Pharmacol. Ther. 233, 108026. 10.1016/j.pharmthera.2021.108026 34718071

[B177] Xu D. Wu D. Qin M. Nih L. R. Liu C. Cao Z. (2019). Efficient Delivery of Nerve Growth Factors to the Central Nervous System for Neural Regeneration. Adv. Mater 31 (33), e1900727. 10.1002/adma.201900727 31125138

[B178] Yang J. Wang L. Huang L. Che X. Zhang Z. Wang C. (2021). Receptor‐targeting Nanomaterials Alleviate Binge Drinking‐induced Neurodegeneration as Artificial Neurotrophins. Exploration 1 (1), 61–74. 10.1002/exp.20210004 PMC10291571 37366469

[B179] Yin L. Ding J. Fei L. He M. Cui F. Tang C. (2008). Beneficial Properties for Insulin Absorption Using Superporous Hydrogel Containing Interpenetrating Polymer Network as Oral Delivery Vehicles. Int. J. Pharm. 350 (1-2), 220–229. 10.1016/j.ijpharm.2007.08.051 17976932

[B180] Ying X. Wang Y. Liang J. Yue J. Xu C. Lu L. (2014). Angiopep-conjugated Electro-Responsive Hydrogel Nanoparticles: Therapeutic Potential for Epilepsy. Angew. Chem. Int. Ed. Engl. 53 (46), 12436–12440. 10.1002/anie.201403846 25044856

[B181] Zain-ul-Abdin A. Wang L. Yu H. Saleem M. Akram M. Khalid H. (2017). Synthesis of Ethylene Diamine-Based Ferrocene Terminated Dendrimers and Their Application as Burning Rate Catalysts. J. Colloid Interface Sci. 487, 38–51. 10.1016/j.jcis.2016.10.001 27743544

[B182] Zatta P. Drago D. Bolognin S. Sensi S. L. (2009). Alzheimer's Disease, Metal Ions and Metal Homeostatic Therapy. Trends Pharmacol. Sci. 30 (7), 346–355. 10.1016/j.tips.2009.05.002 19540003

[B183] Zhang C. Pan D. Luo K. She W. Guo C. Yang Y. (2014). Peptide Dendrimer-Doxorubicin Conjugate-Based Nanoparticles as an Enzyme-Responsive Drug Delivery System for Cancer Therapy. Adv. Healthc. Mat. 3 (8), 1299–1308. 10.1002/adhm.201300601 24706635

[B184] Zhang G. Zhang L. Rao H. Wang Y. Li Q. Qi W. (2020). Role of Molecular Chirality and Solvents in Directing the Self-Assembly of Peptide into an Ultra-pH-sensitive Hydrogel. J. Colloid Interface Sci. 577, 388–396. 10.1016/j.jcis.2020.05.087 32497920

[B185] Zhang L. Zhang F. Weng Z. Brown B. N. Yan H. Ma X. M. (2013). Effect of an Inductive Hydrogel Composed of Urinary Bladder Matrix upon Functional Recovery Following Traumatic Brain Injury. Tissue Eng. Part A 19 (17-18), 1909–1918. 10.1089/ten.TEA.2012.0622 23596981 PMC3726021

[B186] Zhang M. Asghar S. Tian C. Hu Z. Ping Q. Chen Z. (2021). Lactoferrin/phenylboronic Acid-Functionalized Hyaluronic Acid Nanogels Loading Doxorubicin Hydrochloride for Targeting Glioma. Carbohydr. Polym. 253, 117194. 10.1016/j.carbpol.2020.117194 33278970

[B187] Zhang P. Hu L. Yin Q. Zhang Z. Feng L. Li Y. (2012). Transferrin-conjugated Polyphosphoester Hybrid Micelle Loading Paclitaxel for Brain-Targeting Delivery: Synthesis, Preparation and *In Vivo* Evaluation. J. Control. Release 159 (3), 429–434. 10.1016/j.jconrel.2012.01.031 22306333

[B188] Zhang Y. Yang H. Wei D. Zhang X. Wang J. Wu X. (2021). Mitochondria‐targeted Nanoparticles in Treatment of Neurodegenerative Diseases. Exploration 1 (3), 1. 10.1002/exp.20210115 PMC10191038 37323688

[B189] Zhang Z. Ji Y. Lin C. Tao L. (2021). Thermosensitive Hydrogel-Functionalized Gold Nanorod/mesoporous MnO2 Nanoparticles for Tumor Cell-Triggered Drug Delivery. Mater. Sci. Eng. C 131, 112504. 10.1016/j.msec.2021.112504 34857290

[B190] Zhao F. Yao D. Guo R. Deng L. Dong A. Zhang J. (2015). Composites of Polymer Hydrogels and Nanoparticulate Systems for Biomedical and Pharmaceutical Applications. Nanomaterials 5 (4), 2054–2130. 10.3390/nano5042054 28347111 PMC5304774

[B191] Zhao Q. Zhang S. Wu F. Li D. Zhang X. Chen W. (2021). Rational Design of Nanogels for Overcoming the Biological Barriers in Various Administration Routes. Angew. Chem. Int. Ed. 60 (27), 14760–14778. 10.1002/anie.201911048 31591803

[B192] Zhou T. Xiao C. Fan J. Chen S. Shen J. Wu W. (2013). A Nanogel of On-Site Tunable pH-Response for Efficient Anticancer Drug Delivery. Acta Biomater. 9 (1), 4546–4557. 10.1016/j.actbio.2012.08.017 22906624

